# Abstracts from the 11th International Conference for Healthcare and Medical Students (ICHAMS): Virtual. 10-12 February 2022

**DOI:** 10.1186/s12919-022-00236-9

**Published:** 2022-10-27

## Plenary Session

### A1: Sleep, Suicide, and Major Depressive Disorder: A CAN-BIND1 Study

#### Alyssa Francis^1^, Sidney Kennedy^2^, Susan Rotzinger^2^, Amanda Ceniti^2^

##### ^1^Royal College of Surgeons in Ireland, School of Medicine, Dublin, Ireland, ^2^Department of Psychiatry, University of Toronto, Toronto, ON, Canada

###### **Correspondence:** Alyssa Francis

**Introduction**

In community samples, 30% of adults suffer from insomnia, with up to one-third experiencing co-occurring suicidal ideation. Furthermore, among people with major depressive disorder (MDD), 75% report insomnia, indicating a correlation between depression, sleep disturbances, and suicide risk. Previous studies have shown that addressing insomnia in patients with MDD decreases suicidal ideation. However, the relationship between insomnia and suicidal ideation has not yet been examined as a predictive measure of treatment response. We hypothesized that suicidal risk, suicidal ideation, and insomnia directly correlate with MDD severity, and are negative predictors of treatment response/remission.

**Methods**

Patients with MDD (n=211) recruited from 6 outpatient centres in Canada were treated with escitalopram for 8 weeks and evaluated at 8 and 16 weeks for clinical and functional response/remission (Montgomery-Asberg Depression Rating Scale [MADRS]). Responders continued escitalopram, while non-responders received adjunctive aripiprazole for a further 8 weeks. Insomnia and suicidal ideation were evaluated at weeks 0, 8, and 16, while suicidal risk was evaluated at baseline. Linear regression modelling and chi-square tests were used to evaluate the relationship between variables, while controlling for age and gender.

**Results**

Individuals with both moderate-to-severe suicidal ideation and insomnia scores had significantly higher MADRS scores at baseline, than those with low scores in at least one measure (p<0.001). Binomial regression indicated moderate-to-severe suicidal risk at baseline is predictive of both response (p=0.003) and remission (p<0.001) at week 8. In the aripiprazole group, those who remitted at week 16 demonstrated significantly lower suicidal ideation scores at week 8 than non-remitters (p=0.015), indicating level of suicidal ideation prior to initiating aripiprazole treatment is correlated to remission. For both insomnia and suicidal ideation, a greater decrease in scores at 8 and 16 weeks significantly correlated with response/remission.

**Conclusion**

Results have the potential to aid in developing personalized MDD drug prescription practices.

## Oral Session

### O1: Deprivation and mortality in 0 to 17 years old Russian youth

#### Anastasia Zelenina^1^

##### ^1^National Medical Research Center for Therapy and Preventive Medicine Russian Federation, Moscow, Russia

**Background**

According to numerous studies, the relationship has been established between children's health and deprivation. The socio-economic status of parents has a strong influence on children's health. To date, the influence of environmental deprivation on the health of children and adolescents remains poorly understood in Russia.

**Methods**

All data were taken from Federal State Statistics Service for 2010. The principal component analysis was used to create the index that includes 17 indicators reflecting the socio-economic and environmental (natural disasters, air quality) deprivation at the regional level in Russia. The deprivation index levels were divided into four quantiles. The deprivation effect was assessed by comparing four quantiles. The first quantile is the least deprived population, the fourth quantile is the most deprived population. A negative binomial regression was used to establish the relationship between all-cause mortality rate (per 100,000 person-years) at the age of 0-17 years (both sexes/males/females) and deprivation. Spearman's correlation is used to establish an association between deprivation and individual causes of death at the age of 0-17 (both sexes/men/women): respiratory diseases (pneumonia), external causes (suicides and traffic accidents).

**Results**

The risk of death of the entire population aged 0-17 years increases by 22% (95% CI 1.04-1.43, p=0.014) in Q4 compared to Q1, among men and women mortality increases by 20% (95% CI 1.02-1.4, p=0.028) and 25% (95% CI 1.1-1.5, p=0.011) respectively. A positive relationship was established between deprivation and the level of death from deliberate self-harm (suicide) at the age of 0-17 years (ρ=0.42, p<0.001) and individual components of the index - social (ρ=0.27, p=0.003), economic (ρ=0.32, p=0.014) and environmental (ρ=0.31, p=0.004).

**Discussion**

In the index, socio-economic and environmental indicators are combined, reflecting the levels of deprivation and allowing a more in-depth study of the impact of the socio-economic and environmental conditions on children's health in Russia.

### O2: Effect of Superior Capsular Reconstruction with Bursal Acromial Reconstruction on Muscle Excursion During Glenohumeral Abduction

#### Michael Labib^**1**^, Farid Amirouche^2^, Sonia Pradhan^2^, Roberto Leonardo Diaz^2^, Aimee Bobko^2^, Jason Koh^3^

##### ^1^Royal College of Surgeons in Ireland, Ireland, ^2^University of Illinois, United States, ^3^Northshore University Healthsystem

###### **Correspondence:** Michael Labib

**Introduction**

The use of superior capsular reconstruction (SCR) with bursal acromial reconstruction (BAR) is a promising combination of arthroscopic techniques for a subset of patients with massive irreparable rotator cuff (RC) tears. Optimal surgical techniques for RC tears can be identified by comparing muscle excursions measured during surgery. In the following study, the change in shoulder muscle excursion is analyzed after the use of SCR with BAR to treat complete supraspinatus tears.

**Methods**

Six cadaveric shoulders were analyzed using a biomechanical loading apparatus. Digitized points at the origins and insertions of each muscle were tracked while being loaded with free-hanging weights. The muscle excursion of each muscle was measured at 0, 30, 45, 60, and 90° of glenohumeral abduction under four different conditions: (1) intact RC, (2) partial supraspinatus tear, (3) complete supraspinatus tear, and (4) SCR + BAR. Muscle excursions at the four conditions were then compared using Anova and post-hoc Tukey HSD testing performed on Microsoft Excel (version 2107) and Astasa (Vasavada, 2016) respectively.

**Results**

After SCR with BAR, subscapularis muscle excursion was found significantly greater (post-hoc Tukey HSD test; p < 0.01) than when the RC was intact, partially torn, or completely torn. The muscle excursions of supraspinatus (Anova test; p = 0.760), deltoid ( p = 0.336), teres minor ( p = 0.693), and infraspinatus ( p = 0.407) muscles were not significantly different between the four conditions.

**Discussion**

This study proves that SCR with BAR may be used to preserve deltoid and most RC muscle excursions after a massive, irreparable RC tear. Only subscapularis muscle excursion may increase after this surgical combination when compared to an intact, partially torn, or completely torn RC. Therefore, SCR with BAR may increase subscapularis muscle compensatory function during glenohumeral abduction from 0-90°.

### O3: Loss of NFE2L3 protects against inflammatory colorectal cancer through modulation of the tumour microenvironment

#### James Saliba, Mohamad Omar Diab

##### Lady Davis Institute for Medical Research, McGill University, Goodman Cancer Research Centre

###### **Correspondence:** Mohamad Omar Diab

We investigated the role of NFE2L3, a member of the CNC transcription factor family, in inflammation-induced colorectal cancer. Our studies revealed that Nfe2l3 knockout mice display a significant reduction in both tumour size and numbers compared to wild type animals, with Nfe2l3+/- mice exhibiting an intermediate phenotype. Nfe2l3 deficient animals also develop less severe inflammation. We performed RNA-seq analysis of normal and tumour tissue and used CIBERSORT to profile immune cell infiltrates. CIBERSORT predicted a decrease in mast cell numbers in Nfe2l3-/- mice that was confirmed by toluidine blue staining. Concomitantly, the transcript levels of IL33, a mediator of mast cell activation, were also reduced in colons of Nfe2l3 knockout animals. We performed gene set enrichment analysis identifying significant changes in the RAB secretion pathway in Nfe2l3 deficient animals. We confirmed induction of Rab27a, Rab27b, Myrip and Sytl4 transcripts in Nfe2l3-/- mice. Using digital spatial profiling, we found that Nfe2l3-/- mice presented elevated Treg counts and immune checkpoint signatures in the tumour microenvironment. We further validated these data by CD3 and FOXP3 staining revealing a significant increase in Tregs in the colon of Nfe2l3 knockout mice. Our studies uncovered a novel link between NFE2L3 and colitis 39 associated tumorigenesis, showing that loss of Nfe2l3 leads to a decrease in mast cell recruitment, inflammation and tumorigenesis coupled with an increase in the presence of immunosuppressive Tregs. The observed changes in the tumour microenvironment during colon cancer development may be exploited for targeted therapy in CRC patients.

## Poster Session

### P1: A Hybrid Systematic Review of Randomised Controlled Trials Investigating Interventions Designed to Increase Response Rates to Postal and Electronic Questionnaires

#### Arya Pezeshki, An Vu, Dana Al Mubarak, Catherine Moran, Frank Doyle

##### Royal College of Surgeons in Ireland

###### **Correspondence:** Arya Pezeshki

**Introduction**

Surveys are an important technique for gathering information in research. There has been a trend of decline in response rates to surveys. A decrease in response rates to surveys decreases the accuracy of deductions about populations. The decreasing accuracy of surveys in assessing populations is a concern for researchers. This review aims to systematically review the extent to which various factors affect response rates to postal and electronic questionnaires, updating a previous Cochrane review.

**Methods**

A previous Cochrane review provided the search strategy. A hybrid umbrella review and systematic review methodology was conducted through a search for systematic reviews published since 2008 and extraction of studies included in these review reference lists, and an additional comprehensive search for individual randomized controlled trials published over the previous 2-5 years. CINAHL, PsycInfo, and MEDLINE electronic databases were searched. The search terms and hybrid strategy were adapted from Edwards et al. (2009) and Doyle et al. (2021), respectively. In addition, Google Scholar’s ‘cited by’ function was utilised to search for other relevant studies not found using the aforementioned databases. References were downloaded and managed in EndNote X9 software and duplicates removed. Two independent reviewers screened study titles and abstracts for eligibility, and any discordance was discussed and adjudicated by a third reviewer.

**Results**

The general factors identified across 317 papers included monetary incentives, non-monetary incentives, the size of an incentive, the conditionality of an incentive, length of survey, size of font, personalisation, and reminders. All of these factors were found to have some ability of increasing response rates.

**Discussion**

This review updates the evidence for the ability of various factors to increase response rates and is supportive of its findings. Future research could conduct further network meta-analysis to directly and indirectly compare the interventions on their relative effectiveness for increasing response rates.

### P2: A Rare Case of Erosive Arthritis

#### Ioana-Irina Rezus^1^, Vasile-Claudiu Mihai^1^, Elena Rezus^1,2^

##### ^1^”Grigore T. Popa” University of Medicine and Pharmacy Iași, Romania ^2^Clinical Rehabilitation Hospital Iași, Romania

###### **Correspondence:** Ioana-Irina Rezus

**Introduction**

Multicentric reticulohistiocytosis (MRH) is an extremely rare cause of destructive inflammatory arthritis involving both small, as well as larger joints. Only 300 cases have been reported around the world until now.

**Case Report**

We report the case of a 40-year-old Caucasian female with a family history of neoplasia who was referred to our service with a two-month history of inflammatory joint pain and morning stiffness. She also presented xerostomia, xerophtalmia, and skin changes. On examination, the patient had inflammatory arthritis, mainly involving the peripheral joints, sacroiliac joint pain, and numerous papulonodular mucocutaneous lesions, including periungual “coral beads”. She also displayed dermatomyositis-like rash on the upper thorax, as well as yellowish papular plaques on the lower eyelids (suggestive of xanthelasma palpebrarum). Imaging tests revealed erosive arthritis with synovitis and tenosynovitis, as well as sacroiliac joint changes. The endoscopic examination of the upper airways identified papulonodular lesions in the nasal vestibule, pharyngeal mucosa, supraglottic larynx, and arytenoid mucosa. The capillaroscopic examination described papulonodular lesions with a vascular center intercalated with areas of normal capillary density and morphology. The skin biopsy was suggestive of MRH, revealing a mononuclear cell infiltrate and isolated lymphocytes with frequent giant multinucleated cells located between the dermal collagen fibers. On immunohistochemistry, the mono- and multinucleated giant cells were positive for CD68 and negative for S100. She tested positive for HLA-B*07 (Human Leukocyte Antigen B*07) and HLA-B*08, ANA (antinuclear antibodies), RF (rheumatoid factor), anti-Ro52, anti-SSA/Ro, and anti-SSB/La antibodies. The patient exhibited favorable outcomes under Methotrexate, then Leflunomide. However, she displayed worsening clinical symptoms while under Azathioprine.

**Discussion**

To our knowledge, this is the first case of MRH to exhibit positive HLA-B*07 together with HLA-B*08. Mainly due to the rarity of the disease, MRH continues to pose diagnostic and therapeutic challenges for clinicians as the literature leaves many unknowns regarding the optimal treatment.

### P3: A retrospective cohort study comparing the outcomes of conservative versus operative fixation of distal radius fractures in children

#### Taiba Aladraj^1^, Ahmed Keshta^1^, Anas Zeidan^1^, Mohammad Abousaleh^1^, Nitya Kumar^1^, Iftikhar Mukhtar^2^

##### ^1^Royal College of Surgeons in Ireland (Busaiteen, Bahrain) – School of Medicine, Bahrain, ^2^Department of pediatric orthopedic surgery, Bahrain Defense Force (BDF) Hospital, Riffa, Bahrain

###### **Correspondence:** Ahmed Keshta

Distal radius fractures (DRFs) are common orthopedic injury accompanied by suffering and substantial health care costs. Statistics show a significant increase in the incidence of DRFs in children and adolescents. In pediatric population, DRFs represents 25% of all fractures with the significant incidence in the age group 10-14 years. We studied all children presented with distal radius fracture to the emergency department from January 1, 2015, to February 1, 2022, as they were retrospectively enrolled in the study. A total of 176 patients were included. Seventy-seven patients were conservatively managed with cast immobilization (“non-operative” group) in comparison to ninety-nine patients who were surgically managed (“operative” group) with either percutaneous pinning (n=56) or flexinail (n=43). Fewer patients underwent physiotherapy in the operative group with 14 (25.0%) patients for percutaneous pinning and 7 (16.3%) patients for flexinail vs 31 (40.3%) patients in the non-operative group (p<0.015). There were statistically significant differences in radial inclination (p<0.001) between conservative vs Percutaneous pinning (22.22±2.86 vs 18.76±3.33 degrees ) and percutaneous pinning vs flexinail (18.76±3.33 vs 22.37±3.44 degrees). likewise, there was a significant difference found in ulnar variance between conservative vs percutaneous pinning (-0.45±2.14 vs -1.47±1.93, p=0.012) and conservative and flexinail (-0.45±2.14 vs -1.59±1.90, p=0.009. There were total of 25 documented complications. 19 (19.8%) complications occurred in the non-operative group vs. 5 (7.2%) and 1 (2.3%) complications in percutaneous pinning and flexinail groups, respectively (p=0.003). The most common complication in the non-operative group was loss of reduction while in cast and subsequent need for surgical intervention, 10 of these patients underwent percutaneous pinning whereas underwent were 9 fixed my flexinail. This study illustrated an overall similar success between the surgical and the conservative treatments of distal radius fractures in children. However, because of the relatively small sample size, the evaluation was subject to reporting bias.

### P4: A survey of immunization practices in patients with congenital heart disease

#### Gabrielle Sanatani^1^, Sonia Franciosi^2,3^, Jeffrey Bone^3^, Brynn Dechert^4^, Kevin Harris^2^, Manish Sadarangani^5^

##### ^1^Royal College of Surgeons in Ireland, Ireland, ^2^Division of Cardiology, Department of Pediatrics, University of British Columbia, BC Children’s Hospital Heart Centre, Vancouver, Canada, ^3^British Columbia Children’s Hospital Research Institute, Vancouver, Canada, ^4^University of Michigan Congenital Heart Center, Michigan, United States, ^5^Division of Infectious Diseases, Department of Pediatrics, University of British Columbia, Vancouver, Canada

###### **Correspondence:** Gabrielle Sanatani

**Introduction**

Congenital heart disease (CHD), the most common congenital anomaly, often presents in neonates. Because of the risk of fever following immunization and potential exacerbation of CHD, healthcare providers may consider deferring infant immunizations until the CHD has been repaired. The aim of this study was to gain an understanding of perceived risk of immunizations in those who provide healthcare to children with particular heart conditions.

**Methods**

A survey outlining six hypothetical scenarios assessing immunization recommendations was distributed to healthcare providers internationally. The lesions selected represented clear categories in which a fever or reduced oral intake may be a significant hemodynamic challenge for an infant and responses were compared between the different scenarios.

**Results**

A total of 142 survey responses were received; majority from pediatric cardiologists (n= 98; 69%) and nurse practitioners (n= 27; 19%) located in the United States (n=77; 54%) or Canada (n=53; 37%) working in academic teaching hospitals (n=133; 94%). Most were strongly in favour of vaccinations (n=107; 75%) and less likely to proceed with the first immunization in infants with structural heart disease compared to channelopathy (RR 0.80, CI: 0.73-0.87; p<0.001). In the scenario describing an infant with manifest Brugada type I ECG, only 40% would proceed with immunization as normal. Special precautions following the immunization included longer duration of observation (19%) and administering prophylactic antipyretic medication (92%).

**Discussion**

Respondents were 20% more likely to defer immunizations in the presence of treatable structural heart disease as compared to channelopathy despite the lack of evidence supporting deferring immunizations in children with structural heart disease. Most were cautious in their response to the scenario involving Brugada syndrome, indicating awareness of the risk of hemodynamic instability in the event of a fever. The majority of respondents still strongly recommend immunizations in this population.

### P5: A systematic review and meta-analysis of cognitive impairment in chronic obstructive pulmonary disease

#### Austin B. Auyeung^1^, Tan Hoong Wei^1^, Ramsey Keilani,^1^ Trisha Neelakant^1^, Megan Blackburne^2^, Frank Doyle^3^, Catherine Moran^3^

##### ^1^Royal College of Surgeons in Ireland, ^2^Psychology Department of Ireland’s Health Services, ^3^Department of Health Psychology, Royal College of Surgeons in Ireland, Dublin, Ireland

###### **Correspondence:** Austin B. Auyeung

**Introduction**

Chronic obstructive pulmonary disease (COPD) is an increasingly important health issue characterised by progressive chronic airflow limitation and accompanying comorbidities. In 2020, COPD was estimated to be the third leading cause of death and fifth leading cause of disability worldwide. COPD patients are typically counselled in smoking cessation, self-management practices, prescribed multiple medications, inhalers and at the later stages, oxygen. Cognitive impairment associated with COPD may impact a patient’s understanding of new information, medication adherence and self-management. The prevalence of cognitive impairment in those with COPD should be further elucidated to understand its impact on patients and the healthcare system.

**Methods**

PubMed, PsycInfo, CINAHL, Scopus and Embase databases were searched for peer-reviewed articles recording COPD and cognitive impairment. Two reviewers independently screened and assessed references according to the inclusion criteria and any discrepancies were reviewed by a third. Included articles were assessed using the Joanna Briggs Institute Critical Appraisal Tool to determine the quality of each study.

**Results**

The initial search identified 7956 studies. After removal of duplicates, 73 full text articles were assessed for eligibility and 40 studies were included in the qualitative analysis. Preliminary results suggest increased prevalence of cognitive impairment in COPD patients compared to controls.

**Discussion**

Although it has been reported that patients with COPD are more likely to be cognitively impaired, the prevalence is uncertain due to differences in assessment tools and cutoff scores across studies. Our extensive search encompasses these studies, to be considered for further data analysis. A meta-analysis will be conducted to better quantify the overall prevalence of cognitive impairment in COPD patients across the literature. Limitations of our study include potential confounding of common concomitant conditions to COPD such as anxiety, depression, obstructive sleep apnea and dyspnea. The wide array of study designs may increase heterogeneity and introduce bias.

### P6: Assessment of Knowledge, Attitude & Perception towards COVID-19 Vaccine among the rural and urban population of Navi Mumbai: An alarming scenario

#### Akatya Vidushi Sinha, Madhavi Jogesh Mankar

##### MGM Medical College, India

###### **Correspondence:** Akatya Vidushi Sinha

**Introduction**

The launch of the COVID-19 vaccine has been an accelerated program, with the vaccine going to market merely nine months after the discovery of the virus. Vaccine hesitancy may become an important challenge in the immunization campaign against COVID- 19 and thus it is important to understand the current views of the population to bust various myths and impart correct information where necessary. Hence, this study was done to assess community knowledge, attitudes, and perceptions about COVID-19 vaccinations in order to address all barriers to vaccine acceptance in the community.

**Methodology**

A cross-sectional comparative study was conducted between the urban and rural populations with the help of pre-designed and pre-structured questionnaires. Data were collected from 205 subjects from the urban population and 242 subjects from the rural population with the help of Google Forms.

**Results**

97.1% of the urban population were aware of the Arogya Setu app of which 80% were using it while 87.6% of the rural population were not aware of the Arogya Setu app. 60.3% of the rural population had the perception that the Covid-19 vaccine can be eradicated without the vaccine whereas 77.1% of the urban population had the opposite perception.

**Discussion**

Most of the urban population had good knowledge about the Covid 19 vaccine with a positive attitude of accepting the vaccine as the most important preventive measure of prevention and control of the Covid 19 pandemic as compared to the rural population.

### P7: Benchtop Analysis of 2D and 3D In-Vitro Devices

#### Gerges Abdelsayed, Aamir Hameed, Andrew Malone

##### Royal College of Surgeons in Ireland, Dublin, Ireland

###### **Correspondence:** Gerges Abdelsayed

**Introduction**

Cardiovascular disease is the number one cause of death globally, driving demand for the development of cost-effective management. Before new management may be brought to market, it must first demonstrate safety and efficacy. Since the use of humans and animals to test a drug may not be an ethically viable option, in-vitro alternatives may be used. Previously, 2-dimensional in-vitro cardiac models have predominated, but due to technological advancements, 3-dimensional models are emerging. This paper aims to provide an overview of 2D and 3D in-vitro cardiac models and discuss their advantages and limitations.

**Methods**

The MedlineOVID database was used to complete a systematic review of internationally published literature on 2D and 3D in-vitro cardiac models as of March 2021. The following MeSH terms were used: 2D OR 3D AND In-Vitro AND Cardiac. All abstracts were screened. Full texts of selected abstracts were reviewed in detail.

**Results**

Analysis of the literature has shown that 2D models are inexpensive, easy to develop, and well established. They are useful for studying cardiac myocytes at the cellular level but are unable to recapitulate the heart’s 3D microenvironment. For this, 3D models are needed. However, these models are not yet standardized or fully established, and are difficult to develop and use.

**Discussion**

Until further research can be conducted to increase the accessibility and practicality of 3D devices, the decision to use a 2D device versus a 3D device should be made in the context of the particular research project that the device is needed for.

### P8: Biological therapy in infectious diseases – the COVID-19 experience

#### Maria Sorana Manuca, Ioana Onofrei

##### Grigore T. Popa University of Medicine and Pharmacy, Romania

###### **Correspondence:** Maria Sorana Manuca

**Introduction**

Anti-cytokine monoclonal antibodies could in some cases even be a rescue therapy in severe infections that do not respond to antibiotic therapy. Interferon treatment in infectious pathology may be considered the best example of cytokine therapy, but it has not been used for its immunomodulatory effects, but for its direct antiviral action.

**Methods**

The use of monoclonal antibodies in infectious pathology within the Iasi Infectious Diseases Hospital is limited to the period 2020 - 2021, during the COVID-19 pandemic. Tocilizumab (IL-6 receptor antagonist) was administered at an average dose of 530 mg / patient, in a number of 1067 patients (631 men and 436 women) and Anakinra (IL-1 receptor antagonist) at an average dose of 615 mg / patient in 318 patients (168 men and 150 women).

**Results**

Off label administration of monoclonal antibodies is limited to severe cases of COVID -19 pneumonia (based on imagistic results) associating clinical manifestations of respiratory failure with need of oxygen supplementation, invasive/non – invasive mechanical ventilation, persistent fever, and cytokines storm (revealed by increase of inflammatory markers, LDH, ferritin, D-dimers, etc). Therapeutic response was very variable, depending on various demographic factors (age, gender) and comorbidities (metabolic, cardiovascular, neoplasms, autoimmune disorders).

**Discussion**

Although the role of cytokines in infectious diseases is already established, therapeutic immumodulation in infectious pathology has only been used during the COVID-19 pandemic. Significantly elevated levels of certain cytokines have been detected in patients with severe SARS-CoV-2 infection. There is no doubt that IL-6 plays a key role in development and amplification of cytokine storms that underlie lung and systemic lesions caused by SARS-CoV-2. In this context, administration of IL-6 receptor antagonists (tocilizumab) has been an effective approach for treatment of severe SARS-CoV-2 infections.

### P9: Can we improve laparoscopic skills by practical activities?

#### Agata Kaczmarek, Patrycja Sienkiewicz-Sosnowska

##### Department of Pediatric Surgery, Traumatology and Urology, Poznań University of Medical Sciences, Poznan, Poland

###### **Correspondence:** Agata Kaczmarek

**Introduction**

While learning laparoscopic procedures, the physician must face the difficulties specific to this method, such as two-dimensional vision, disturbed eye-hand coordination, and lack of touch sense control. We examined the impact of individual abilities and different types of training on the performance of laparoscopic procedures on box trainers.

**Methods**

Students who had no previous experience in laparoscopy participated in the study. They took part in Minnesota Manual Dexterity Test (MMDT). Then they were divided randomly into three groups based on the results of MMDT. 1st group had a 3-hours laparoscopic training, in which each participant could prove himself as the operator and assistant. The 2nd group was performing visual-manual tasks using a phone application. The 3rd group took part in the final test without prior preparation. The final test included assignments of transferring sponges using the laparoscopic trainer and cutting a latex glove. We checked the differences in the final test between groups using the statistic ANOVA rang Kruskal- Wallis test.

**Results**

There was a statistically significant difference between the groups in the results of the sponge transfer test and the glove cutting speed. No correlation was observed between the perforations during the cutting latex glove and the cutting length.

**Discussion**

The laparoscopic trainer - also self-built - is a valuable tool for laparoscopic training. The use of arcade smartphone games contributes to the increase of laparoscopic psychomotor skills. These tools are handy during the COVID-19 pandemic. They enable the continuous improvement of laparoscopic skills without the need for contact classes.

### P10: Cancer spares no one: a lifetime fighter

#### Alexandra Condurat, Andra Diana Tanasa, Alice Colescu

##### Grigore T. Popa University of Medicine and Pharmacy Iasi, Romania

###### **Correspondence:** Alexandra Condurat

**Background**: Hepatocellular carcinoma is the most common type of primary liver cancer with a survival rate of approximately 6 to 20 months. Moreover, liver cancer is the 6th most common cancer type in men found most often in people with chronic liver diseases, such as cirrhosis caused by B or C virus. This case brings in front of you a 40 year-smoker who suffered from chronic alcoholism, who successfully passes 4 surgeries on 4 primary malign tumors, who was infected with B virus during a transfusion and who survived 14 years after the diagnosis. **Case description**: I.C. was diagnosed in March 2007 with chronic ethanolic hepatitis and hepatic tumor situated in the right lobe. Due to ecography we localized a isodense node between the V and VIII segments. One month later, he benefited from an atipic hepatectomy of VIII segment, a cholecystectomy and biliary drainage. The surgery was a success. Unfortunately, due to the weak sanitary system, he gained during transfusion the B hepatitis virus. 7 years later, in 2014 a hepatocellular carcinoma was found in the VI segment. Another surgery was performed, also with a promising result. Moreover, he started chemotherapy after the intervention. In 2019, I.C. was diagnosed with 2 primary malign tumors in 2 different organs: a broncho-pulmonary neoplasm in the left lobe and another hepatocarcinoma in IV segment. Because it was touching the inferior vena cava, the surgeon could only apply focal heat destruction to the affected area. I.C. passed away on 1st of May 2021. **Conclusion**: We have assisted an impressive fight between the human body and cancer. We can now fully understand the effects of B virus, smoking and alcohol over the liver and lungs. The patient passed over any prognosis and statistics being a true survivor considering the mortality rate of his diagnosis.

### P11: Comparative analysis of prostate-specific antigen serum level and histopathological report in needle biopsy of prostate

#### Karla Mutibaric^1^, Tamara Zelenovic^1^, Tanja Lakic^2^, Aleksandra Ilic^3^

##### ^1^Faculty of Medicine, University of Novi Sad, Serbia, ^2^Assistant Professor at Department of Pathology, Faculty of Medicine, University of Novi Sad, Serbia, ^3^Teaching Assistant at Department of Pathology, Faculty of Medicine, University of Novi Sad, Serbia

###### **Correspondence:** Karla Mutibaric

**Introduction**

The prostate secretes enzyme called prostate-specific antigen (PSA), which has a serum level that increases dependent on alterations in prostate. PSA is used for screening and tracking the relapse of Adenocarcinoma. Given the fact that PSA is related to prostate cancer, the aim of this paper was to establish correlation between certain prognostic parameters, as well as correlation and specificity of PSA among men with cancer verified by a core needle biopsy procedure.

**Methods**

The study was retrospective and pathohistological documents from a one year interval, from November 2018 to November 2019, were taken into consideration. The analyzed documents were obtained from the Center for Pathology and Histology of Clinical Center of Vojvodina. The selection of patients was done by a clinical doctor based on PSA levels and physical examination.

**Results**

Out of a total of 181 patients, 96 men (53%) were diagnosed with cancer, 8 (4.4%) had PIN alterations and 77 (42.6%) had benign hyperplastic alterations of epithelium. The mean age of examinees was 69.7 and the age span was 52-91 years old.

**Discussion**

The PSA parameter used to evaluate patients' condition was age adjusted. PSA has shown sensitivity of 100% in the age group of men 50-59 years old, 97.6% in group 60-69 years and in group of men older than 70 years, the sensitivity of PSA was 84.3%. However, when it comes to benign hyperplasia, there is no regularity to be detected.

### P12: Comparative Effectiveness of Closed Reduction with Percutaneous Pinning and Open Reduction with Internal Fixation in Operative Management of Pediatric Type III Supracondylar Fractures

#### Mohammad Abousaleh^1^, Anas Zeidan^1^, Ahmed Keshta^1^, Taiba Aladraj^1^, Nitya Kumar^1^, Iftikhar Mukhtar^2^

##### ^1^Royal College of Surgeons in Ireland (Busaiteen, Bahrain) – School of Medicine, Bahrain, ^2^Department of pediatric orthopedic surgery, Bahrain Defense Force (BDF) Hospital, Riffa, Bahrain

###### **Correspondence:** Ahmed Keshta

**Background**

Supracondylar fracture with total displacement is classified as Gartland Type 3. The operative management for this type of fracture can be in the form of Closed Reduction with Percutaneous Pinning (CRPP) or Open Reduction with Internal Fixation (ORIF).

**Aim**

The study aims to determine whether CRPP or ORIF led to smaller changes in the Baumann’s angle, the carrying angle, loss of motion, and complication when treating pediatric supracondylar fractures.

**Methods**

In a retrospective cohort design, pediatric patients presenting with supracondylar fractures at a tertiary care hospital in Bahrain between March and October of 2021, were enrolled. The collected data included: age, gender, nationality, mechanism of injury, neurovascular status, type of surgery performed, follow-up period, range of motion, complications, Baumann’s angle, carrying angle, and loss of motion were collected. The change in Baumann’s angle, carrying angle, and reduction sufficiency were compared to the literature using Flynn’s criteria for supracondylar fractures.

**Results**

This paper included the records of 60 patients with supracondylar fractures. Twenty-eight patients underwent CRPP (Group A), whereas 32 underwent ORIF (Group B). A statistically significant difference (p=0.037) between group A and group B was noted when the loss of carrying angle scores and the loss of motion scores results were combined to form the final Flynn score. The loss of motion was significantly different between the two groups (p=0.038). The mean loss of carrying angle was significantly different between the two groups, with 5.51 +3.03 degrees for group A and 4.23 +1.85 degrees for group B (p=0.023). The study had only two cases with unsatisfactory ratings, both of which belonged to group A.

**Conclusion**

In pediatric patients presenting with Type 3 supracondylar fractures, when compared to CRPP, ORIF was associated with less loss of motion, less loss of carrying angle, higher overall satisfactory results according to Flynn’s criteria.

### P13: Development of a Comprehensive Deep Learning Neural Network Algorithm for Analysis of Gram-Stained Sputum Smears for Adequacy: An Aid for Developing Countries

#### Manraj Sirohi^1^, Mahima Lall^1^, Swapna Yenishetti^2^, Lakshmi Panat^2^, Ajai Kumar^2^

##### ^1^Armed Forces Medical College, Pune, India, ^2^Centre for Development of Advanced Computing, Pune, India

###### **Correspondence:** Manraj Sirohi

**Introduction**

Sputum is a common laboratory specimen which aids in diagnosing respiratory diseases including Pulmonary Tuberculosis (TB). Gram stain is an easy, cost-effective stain which may be applied to sputum smears to screen out an unsatisfactory sample by applying Bartlett’s Criteria (1979), which determines adequacy, or freedom from contamination, of a sputum sample. In this first study of its kind, we propose a Faster- Region Based Convolutional Neural Network (F-RCNN) based algorithm to verify our hypothesis.

**Hypothesis**

An Artificial Intelligence algorithm can automate the process of determining the adequacy of Gram stained sputum smears using Bartlett’s criteria, reducing time and human error.

**Methods**

The developmental pilot study was carried out by training F-RCNN on 100 Gram Stained Sputum Smear Slides with equal number of adequate and inadequate sputum smears, by using Tensorflow 2 object detection methods on faster_rcnn_resnet50_v1_640x640 pre-trained models, followed by evaluation on Tensorboard. Following evaluation and feedback corrections, the algorithm was then tested on 100 Gram Stained Sputum Smear Slides to determine adequacy as compared to the manual method. Analysis of cell counts, and binary adequacy comparison by a classification table was performed.

**Results**

In 100 Gram Stained Sputum Smear Slides, cell counts matched 76% for Squamous Epithelial Cells, and 91% for Neutrophils. As compared to the manual method, our model reported a Sensitivity of 90.56%, a specificity of 78.72%, and an overall accuracy of 85% in determining whether a slide is adequate or not.

**Conclusions**

The F-RCNN based algorithm was successful in establishing an approach for detection of Squamous Epithelial Cells and Neutrophils in Gram Stained Sputum Smears, and thus determining their adequacy by Bartlett's Criteria.

### P14: Diagnostic Accuracy Of Blood-Based Biomarkers For Pancreatic Cancer

#### Laura E. Kane^1^, Gregory S. Mellotte^2^, Eimear Mylod^3^, Rebecca M. O'Brein^3^, Fiona O'Connell^3^, Ms Croí E. Buckley^3^, Jennifer Arlow^4^, Khanh Nguyen^4^, David Mockler^5^, Aidan D^4^, Barbara M. Ryan^2^, Stephen G. Maher^3^

##### ^1^Trinity College Dublin, Ireland, ^2^Department of Gastroenterology, Tallaght University Hospital, Dublin 24, Ireland, ^3^Department of Surgery, Trinity St. James’s Cancer Institute, St. James's Hospital, Dublin 8, Ireland, ^4^School of Physics and Clinical Optometric Science, Technological University Dublin, Dublin 2, Ireland, ^5^Medical Library, Trinity College Dublin, Dublin 2, Ireland

###### **Correspondence:** Laura E. Kane

**Introduction**: Pancreatic ductal adenocarcinoma (PDAC) has a 5-year survival rate below 5%. CA19-9 is the only FDA-approved blood-based biomarker marker for PDAC diagnosis in current clinical practice, despite having been shown repeatedly to be inaccurate and have poor diagnostic performance. This review aims to assess the reported diagnostic accuracy of all blood-based biomarkers investigated to date in PDAC, by directly comparing individual biomarkers and multi-biomarker panels, both containing CA19-9 and not (novel). **Methods**: A systematic review was conducted in accordance with PRISMA standards. Individualised search strategies for three academic databases (EMBASE, MEDLINE and Web of Science) identified 5,885 studies. After two rounds of screening by two independent reviewers, 250 studies were included. Data were extracted into Google Sheets and assessed for bias using the QUADAS-2 tool. A multivariate three-level meta-analysis with subgroup moderators was run in R (v1.3.959) using AUC values as effect size. **Results**: Based on the three-level meta-analytic model, the pooled AUC value for all multi-marker panels (AUC=0.898, 95% CI: 0.88-0.91) was significantly higher than all single markers (AUC=0.803, 95% CI: 0.78-0.83)(p<0.0001). The pooled AUC value for CA19-9 alone (AUC=0.8473, 95% CI: 0.82-0.87) was significantly lower compared to the multi-marker panels containing CA19-9 (AUC=0.91, 95% CI: 0.90-0.93)(p<0.0001). For the novel markers, the pooled AUC for single markers (AUC=0.79, 95% CI:0.75-0.83) was also significantly lower compared to novel multi-marker panels (AUC=0.87, 95% CI:0.85-0.89) (p<0.0001). **Conclusion**: Overall, multi-marker panels demonstrate significantly higher pooled AUC values than single markers, and this holds true for both CA19-9 and novel datasets. Multi-marker panels containing CA19-9 exhibit the most promising pooled AUC value, with CA19-9 alone performing inferior to novel multi-marker panels. These results suggest that CA19-9 may be best used as an addition to a panel of markers rather than alone, and that multi-marker panels generate the most robust results.

### P15: Do images of cardiopulmonary resuscitation on the Internet reflect gender equality

#### Reeya Gulve^1^, Anuradha Joshi^2^

##### ^1^Undergraduate Student, Bharati Vidyapeeth (Deemed to be University) Medical College, Pune, India, ^2^Professor and Head, Department of Physiology, Bharati Vidyapeeth (Deemed to be University) Medical College, Pune, India

###### **Correspondence:** Reeya Gulve

**Introduction:** Women who suffer an out-of-hospital cardiac arrest, receive bystander cardiopulmonary resuscitation (CPR) less often than men. Understanding whether gender variation persists in the public informational material may present important consideration for future training and public messaging. Given the importance of Internet searches in obtaining information, this study was carried out to assess whether images of CPR on the Internet reflect the gender equality of person experiencing cardiac arrest. **Methods:** A cross-sectional observational study was conducted to analyze publicly available images on the Internet. Keyword was identified by compiling a list of possible words related to CPR namely, Cardiopulmonary resuscitation, CPR, Basic life support, BSL, Heart massage, Cardiac massage, Chest compressions. The most frequently word was identified using Google Trend search, and was selected as the keyword for the study, namely ‘CPR’. Images appearing in Google images searches using the word ‘CPR’ were analyzed for content. Images not related to adult CPR, unclear images, CPR on unisex manikin were eliminated. Images with human symbol (real human photographs, pictures and cartoons) in which sex could be distinguished were classified according to gender of recipient of CPR. **Results:** From total of 744 images retrieved, 629 were excluded. Of the remaining images, only 26 (23%) featured a women receiving CPR as compared to 89 (77%) featured a men receiving CPR. **Discussion:** Images of CPR performed on men and women recipients are notably different in terms of availability. There are limited numbers of images available on Internet featuring CPR performed on women. Improved gender presentation in CPR images can help to close the gap in bystander CPR disparities. This, however, is insufficient. To begin addressing and eventually diminishing gender disparities in CPR, female-specific CPR barriers (e.g. fear about inappropriate touching, misconceptions of women in cardiac arrest) must be specifically addressed in CPR educational material.

### P16: Effect of Bursal Acromial Reconstruction (BAR) on Muscle Excursion During Glenohumeral Abduction

#### Michael Labib^1^, Farid Amirouche^2^, Sonia Pradhan^2^, Roberto Leonardo Diaz^2^, Aimee Bobko^2^, Jason Koh^3^

##### ^1^Royal College of Surgeons in Ireland, Dublin, Ireland, ^2^University of Illinois, United States, ^3^Northshore University Healthsystem, United States

###### **Correspondence:** Michael Labib

**Introduction**

Bursal acromial reconstruction (BAR) is a new and evolving surgical technique for a subset of massive irreparable rotator cuff (RC) tears. Muscle excursion measured during surgery can help differentiate between the surgical alternatives available for irreparable RC tears. This study aims to identify how shoulder muscle excursion is affected by the treatment of complete supraspinatus tear using BAR.

**Methods**

Six cadaveric shoulders were examined using an experimental loading apparatus. The origin of each shoulder muscle was loaded using a free-hanging weight. Muscle excursions were then measured while the shoulders were held at 0, 30, 45, 60, and 90° of glenohumeral abduction under four different conditions: (1) intact RC, (2) partially torn supraspinatus, (3) completely torn supraspinatus, and (4) after BAR.

**Results**

Subscapularis muscle excursion after BAR was significantly larger (post-hoc Tukey HSD test; p < 0.01) compared to when the supraspinatus was intact, partial torn, or completely torn. Supraspinatus (Anova test; p = 0.684), infraspinatus (p = 0.236), teres minor (p = 0.247), and deltoid (p = 0.482) muscle excursions did not significantly differ between the four conditions.

**Discussion**

Following a complete supraspinatus tear, deltoid and most RC muscle excursion may be preserved using BAR. However, subscapularis muscle excursion may increase after BAR compared to the intact, partially torn, and completely torn RC. This indicates that BAR may increase the compensatory function of the subscapularis muscle during 0-90° of glenohumeral abduction.

### P17: Efficacy and Safety of Adjunctive Dexamethasone Therapy in Adolescents and Adults with Bacterial Meningitis: A Systematic Review of Randomized Controlled Trials

#### Maryam Alarayedh, Ola Alhalwachi

##### Royal College of Surgeons in Ireland (Busaiteen, Bahrain) – School of Medicine Bahrain

###### **Correspondence:** Maryam Alarayedh

**Background:** Controversy around Dexamethasone use in the therapeutic regimen of bacterial meningitis has been a long-standing topic of interest. Conflicting results from multiple studies have made it difficult to determine the exact benefit of this practice. **Methods:** Several online databases (PubMed Central, ScienceDirect, Google Scholar, Wiley Online Library, Academic Search Ultimate, Medline, CINAHL, JAMA Network, and BMJ journal) were searched for RCTs of Dexamethasone use in adolescents and adults with bacterial meningitis published between 1991-2021. The studies' methodological quality was assessed using the Joanna Briggs Institute assessment tool to ascertain data rigour and trial reliability. **Results:** A total of 7 trials including 1438 bacterial meningitis patients (721 Dexamethasone group, 717 control group) were included in our systematic review. Our results established that Dexamethasone was associated with a reduction in mortality. However, three studies had lower mortality in their Dexamethasone group but did not reach statistical significance. Moreover, there was no beneficial effect on neurological sequelae and inconclusive results in hearing loss. **Discussion:** Dexamethasone use in conjugation with antimicrobials proves to reduce the risk of mortality. However, there is no significant beneficial effect on neurological sequelae and an undetermined effect on hearing loss. More trials with larger sample sizes are needed to investigate the efficacy of this treatment in adolescents and adults.

### P18: Emetin as a regulator of cell junctions in breast cancer

#### Diana Grigoreva^1^, Olga Zhukova^2^, Ekaterina Zhidkova^1^

##### ^1^N.N. Blokhin National Medical Research Center of Oncology Russian Federation, ^2^MIREA — Russian Technological University Russian Federation

###### **Correspondence:** Diana Grigoreva

**Introduction**

Metastatic breast cancer (BC) is an aggressive cancer with poor prognosis. Metastatic potential of BC is mainly associated with the failure of cell adhesion and loss of cell junctions. Metastatic BC is characterized by low expression of E-cadherin, key molecule of cell adhesion. Anti-emetic compound Emetin demonstrates anti-cancer activity in solid cancers. In particular, it induces apoptosis and suppress proliferation, migration of BC cells as well as formation of BC spheroids. The aim of the presented study was to evaluate the effect of Emetin of signaling involved in BC metastasis.

**Methods**

Emetin was chosen as potential anti-cancer compound via bioinformatical screening. BC cells of different subtypes (luminal A BC MCF-7, triple negative BC MDA-MB-231 and HER-2-positive BC HCC-1954) were treated with Emetin (0,1 uM). Specific gene expression (SK1, MMP1, MMP9, RHOB, CDH1) was evaluated by quantitative PCR analysis. The level of E-cadherin protein was evaluated by Western blot analysis.

**Results**

We demonstrated 2-fold decrease in CDH1 expression after the treatment of HER2+ BC cells HCC-1954 with Emetin for 24 h. In contrary, in the basal and luminal BC cells it was shown the 1,5-fold increase in E-cadherin expression. However, we did not demonstrate any significant difference in the E-cadherin expression on protein level as well as in the mRNA level of SK1, MMP1, MMP9, RHOB genes.

**Discussion**

We demonstrated that Emetin could prevent the metastasis of triple negative and luminal BC via the increase of the expression of E-cadherin. Work was supported by Russian Science Foundation grant 17-75-20124

### P19: Emotional Intelligence of Undergraduate Medical Students of Public and Private Medical Schools

#### Almas Khattak^1^, Sobia Nasim^2^, Mustafa Qazi^1^, Gul Rukh Iqbal^1^

##### ^1^North West School of Medicine, Peshawar, Pakistan, ^2^Trim Primary Care Centre, Longford Co Meath, Ireland

###### **Correspondence:** Mustafa Qazi

**Objective**

In the era of patient-centered healthcare, medical professionals are better equipped if cognizant of their own as well as patients’ feelings and are emotionally responsive. Emotional Intelligence (EQ) has been related to academic success in medical students and better interpersonal interaction and patient satisfaction in clinical practice. This study hypothesized that there is a difference in the EQ of undergraduate medical students of public and private medical schools.

**Methodology**

A cross sectional study was conducted on undergraduate medical students of public and private medical schools in Peshawar, Pakistan, using a pre-validated 60 item questionnaire distributed into 5 domains: self-awareness, self-regulation, motivation, empathy and social skills. Maximum possible score was 300. Overall mean score of students’ EQ was sum of all individual item scores. The data was analyzed using SPSS version 21.

**Results**

A total of 257 medical students participated (169 from private and 88 from public colleges). The overall minimum EQ score was 55 and maximum 292, with a mean EQ score of 209.11 (SD=39.177). Independent sample t-test revealed that females had a higher EQ score (M=212.53, SD=25.996) as compared to males (M=205.38, SD=49.569) t(180.807)=0.155, p<.001. The students of public medical schools (M=212.14, SD=26.883) had significantly higher EQ than the students of private medical schools (M=207.53, SD=44.241): t(248.979)=1.035, p=.004.

**Discussion**

The results support the hypothesis that the EQ of students of private and public medical schools is different. It is important that both public and private medical institutes prepare doctors of tomorrow with sound EQ right from the outset of their professional careers to deliver emotionally responsive healthcare services.

### P20: Evaluating the Impact of Implementing an Inpatient Addiction Medicine Consult Service for People with Alcohol Use Disorder or Opioid Use Disorder

#### Minatoullah Habaka^**1**^, Kate Lazier^2,3^, Rosanra Yoon^3^

##### ^1^Royal College of Surgeons in Ireland, Dublin, Ireland, ^2^University of Toronto Department of Family and Community Medicine, Toronto, Canada, ^3^Michael Garron Hospital, Toronto, Canada

###### **Correspondence:** Minatoullah Habaka

**Introduction**

Substance use disorder (SUD) affects 6 million Canadians. To ensure best quality of patient care, an inpatient addiction consultation service (ACS) was implemented at a large community hospital in East Toronto to improve care for hospitalized people with alcohol use disorder (AUD) or opioid use disorder (OUD). This study aims to assess the characteristics (demographics, admitting diagnoses, SUDs and treatments) of patients seen by the consult service and to examine the number of encounters prior to and following the implementation of the ACS for the following: 1. Patients prescribed Opioid Agonist Therapy (OAT) for OUD 2. Patients prescribed anti-craving medication for AUD

**Methods**

Characteristics of patients seen during the ACS were assessed using a clinician generated database tracking consultations. The program evaluation study was conducted using administrative data pre- and post- implementation of the ACS to measure initiation and/or continuation of pharmacotherapy for OUD. This was measured from March 1st, 2021, to July 1st, 2021, and compared to baseline use of OAT over a 3-month period in 2020 prior to the ACS.

**Results**

Of 125 patients, 25% were admitted due to opioid complications. Since implementation of the program, 63% of patients with OUD were initiated on OAT. Data analysis shows that there is a 42% increase in encounters where OAT was prescribed after implementation of the service.

**Discussion**

The newly implemented ACS resulted in an increase in OAT; however, due to anti-craving medications being listed as non-formulary medications during the timeframe studied, it was not possible to use hospital EMR data to track use of these medications among hospitalized patients. Further research is required to determine whether patients remain on OAT therapy upon discharge. Future directions include a phase 2 of this study which will examine patient and provider experience of the ACS.

### P21: Exploration of genetic alterations in RAD51B in cancer

#### Azmaeen Zarif^1,2^, Ofer Shapira^1^, Rose Gold^1^, Shu Zhang^1^, Simona Dalin^1^, Rameen Beroukhim^1^

##### ^1^Broad Institute of MIT and Harvard, 415 Main St, Cambridge, MA 02142, United States of America, ^2^University of Cambridge School of Clinical Medicine, Hills Rd, Cambridge, CB2 0SP, United Kingdom

###### **Correspondence:** Azmaeen Zarif

**Introduction**

WGS data from the Pan-Cancer Analysis of Whole Genomes (PCAWG) dataset of 2,658 whole-cancer genomes across 38 tumour types found the most mutated DNA break repair mechanisms (DBRMs) genes to be FANCA, POLE, PRKDC and RAD51B. Samples with mutant DBRMs have a significantly higher structural variants (SVs) rate. RAD51B alterations were found to be the most significant predictor of SV burden. Given RAD51B is implicated in homologous recombination (HR), we attempted to characterise its role in oncogenesis.

**Methods**

We conducted analyses using PCAWG germline and somatic sequencing data. We characterized RAD51B alterations to identify consistent changes suggestive of potential deleterious functions.

**Results**

3791 unique RAD51B mutations were found in 42.02% of donors. The distribution of mutation types differed significantly (compared to all PCAWG genomes; p<0.05) with 86.57% (91.62%) SNVs, 9.34% (5.06%) indels<=200bp, 1.74% (1.86%) CNVs, 1.50% (0.60%) SVs and 0.84% (0.85%) MNVs. RAD51B mutations affected a greater proportion of donors with DNA repair pathway mutations (43.67%) compared to TP53 (39.68%), BRCA2 (12.86%) and BRCA1 (12.24%). RAD51B is predominantly intronic (exon:intron bp ratio=0.00208) and ranks lowest when compared to HR and known cancer genes. The five exonic SNVs observed are significantly more than expected (1.39 [95%CI:1.36-1.41] from n=10,000 random simulations). They are primarily located in the DNA-binding BCDX2-complexRAD51C-interacting subdomain. SV breakpoints are distributed relatively evenly across the gene. CNVs are modally (59.1%) amplificatory (CN>=3). SVs and SNVs are enriched in breast and colorectal tumours, respectively (>15% samples, p<0.0001).

**Discussion**

Amplificatory CNVs alongside a significantly greater number of exonic mutations than expected suggest a consistent novel oncogenic functional role. Mutated RAD51B may facilitate deleterious DNA damage during HR, leading to increased cancer susceptibility. Future work is required to assess whether these alterations lead to elevated rates of rearrangement, including by reproducing these events in breast and colorectal cancer model systems.

### P22: Exploratory analysis of troponin as biomarker of acute ischaemic stroke subtypes in a subset group of patients recruited to the MiND study

#### Jelizaveta Cvetkova, Jorin Bejleri, David Williams, Shona Pfeiffer

##### Royal College of Surgeons in Ireland, Dublin, Ireland

###### **Correspondence:** Jelizaveta Cvetkova

**Introduction**

Stroke is a major common cause of acquired disability and death worldwide. Atrial dysfunction defined by the presence of specific serum biomarkers, ECG findings, or echocardiographic findings, increases the risk of atrial fibrillation, which has been implicated as a key risk factor for ischaemic stroke. There is now a growing body of evidence that suggest that early positive troponin after ischaemic stroke may be independently associated with a cardiac embolic source. We hypothesized that early elevated troponin is a predictor of cardiac source of embolus in patients with embolic stroke subtypes (cardioembolic [CE] and embolic stroke of unknown source [ESUS]) compared with non-cardioembolic subtypes (NCE).

**Methods**

Data was retrospectively extracted from patients recruited to the miRNAs as Novel Diagnostic biomarkers (MiND) study with confirmed diagnosis of acute ischaemic stroke within 12 hours of symptoms onset having also undergone high-sensitivity troponin testing from July 2019 to July 2021. Statistical analysis was carried out using STATA/SE, version 16.0. We identified 43 patients eligible for inclusion.

**Results**

Data were dichotomised as normal troponin (<14ng/L) (n = 27) and elevated troponin (≥14ng/L) (n = 16). Stroke subtypes were classified as per ESUS criteria. Twenty-two patients were identified as having CE, 12 as NCE and 9 as ESUS stroke subtypes. The mean age for each group was 77.5 ±9.2, 66.4 ±3.8 and 62 ±11.4 years, respectively. The unadjusted logistic analysis revealed a positive association between CE stroke subtype and elevated troponin (OR 5.1, 95% CI 1.29-20.17, p = 0.02) and a negative association between NCE stroke subtype and elevated troponin (OR 0.79, 95% CI 0.20-3.21, p = 0.744).

**Discussion**

Our analysis suggests the potential utility of early elevated troponin after ischaemic stroke as a biomarker of CE stroke subtype. The data also reveals that early elevated troponin may indicate increased stroke severity on admission and insular infarct.

### P23: Family Physicians Perspective on Telehealth: A Rural Canadian Explorative Study

#### Gerges Abdelsayed^1^, Meghan Gipson^1^, Natalija Lakic^1^, Joseph Girgis^1^, Nivin Azer^2^

##### ^1^Royal College of Surgeons in Ireland, ^2^Regent Primacy Medical Center Canada

###### **Correspondence:** Gerges Abdelsayed

**Introduction**

Telehealth is a broad term encompassing the delivery of healthcare through telecommunication technology, including video, phone, and messaging platforms. The use of telehealth services has increased dramatically during the COVID-19 pandemic, with a recent analysis by McKinsey and Company finding a 38-time increase in utilization when compared to pre-pandemic levels. Although the overall perception by healthcare consumers has been positive, it is unclear whether healthcare providers feel similarly. This study aims to determine the perspective of Canadian rural family physicians on the increased use of telehealth services.

**Methods**

A 17-item survey was produced and used to assess rural family physicians' utilization and perspectives on telehealth pre- and post-pandemic.

**Results**

6 rural family physicians were interviewed. 5/6 physicians indicated that since the pandemic began, they completed 20+ consultations per month using telehealth. This has continued into 2021 for 4/6 of the physicians. 2 physicians used telehealth 1-9 times per month prior to 2020. All the physicians indicated that they enjoyed Telehealth equally or less than in-person consults and that telehealth has contributed to an equal or increased workload. Many physicians indicated that during the pandemic, their work has become more stressful and less enjoyable, but that Telehealth has been a useful tool in that period.

**Discussion**

This exploratory study has found an increased utilization of telehealth services among family physicians in rural Canada. These doctors have found telehealth to be a useful tool, although some enjoy it less than in-person consultations. More research should be conducted to determine the drawbacks of telehealth consultations, and what can be done to address them.

### P24: Inducing transient activation of the PERK pathway in mouse beta cells

#### Daniel Harrington^1^, Rohit B Sharma^2^, Christine Darko^2^, Rachel E Stamateris^3^, Laura C Alonso^4^

##### ^1^Royal College of Surgeons in Ireland, ^2^Division of Endocrinology, Diabetes and Metabolism, ^3^University of Massachusetts Chan Medical School, ^4^Division of Endocrinology, Diabetes and Metabolism, Weill Cornell Medicine United States

###### **Correspondence:** Daniel Harrington

**Introduction**

Beta cell regeneration is a key goal for future diabetes therapies. Transient activation of the unfolded protein response (UPR) stimulates adaptations which include increased β-cell proliferation. Phosphorylated eIF2α (p-eIF2α) signals activation of the PERK pathway of the UPR. The purpose of this study was to test p-eIF2α chemical activators in murine beta cells towards future studies testing their impact on proliferation.

**Methods**

Islets were harvested from mice and dispersed onto culture plates with a standard glucose-containing culture medium, then incubated for 24 hours before adding 8 microliters of experimental reagent or vehicle control (DMSO). The experimental reagents were Salubrinal (75uM); Sal003 (5, 10, or 50uM); and Guanabenz (50, 100, or 125uM). After an additional 24 hours, cell lysates were collected, separated by polyacrylamide gel electrophoresis, transferred to immunoblot membranes which were incubated sequentially with antibodies and then exposed to radiographs. The intensity of the band signals on the radiographs was quantified using ImageJ. Data analysis was performed using the student’s t test on Microsoft excel, with p value <0.05 as the significance threshold.

**Results**

Sal003 and Guanabenz yielded a concentration-dependent increase in the relative abundance of p-eIF2α to the Actin housekeeping control when compared to that same ratio in the vehicle control condition. For Sal003, concentrations of 5 and 10uM significantly increased p-eIF2α abundance while 50uM did not. 125uM Guanabenz significantly increased p-eIF2α while the lower concentrations did not. Treatment with Salubrinal did not significantly increase phosphorylation of eIF2α in these cultures.

**Discussion**

Sal003 and Guanabenz induced the phosphorylation of a key PERK-pathway UPR mediator, eIF2α, in dispersed murine islet cell cultures when administered at certain concentrations. This suggests that these reagents could be used in future work investigating the mechanisms and pathways regulating UPR influence on beta cell proliferation.

### P25: Investigating dysregulated luminal subtype specific p53 pathway genes in the brain metastatic transcriptome in breast cancer.

#### Michael Labib, Nicola Cosgrove, Leonie Young

##### Royal College of Surgeons in Ireland

###### **Correspondence:** Michael Labib

**Introduction**

Brain metastasis affects 20-30% of women with breast cancer, where it is associated with decreased quality of life and poor prognosis. Current treatment strategies consist of surgery, radiation therapy, and chemotherapy. While this standard of care may provide benefit in the short term, targeting genes within dysregulated pathways in the brain metastatic transcriptome may improve patient prognosis long term.

**Methods**

Previously, luminal subtype-specific p53 pathway dysregulation was identified in breast cancer brain metastases from analysis of RNA sequencing data of patient-matched primary breast tumours and resected brain metastatic tumour samples (N = 90) from a 45 patient cohort.

**Results**

The brain metastatic tumour gene expression for each of the dysregulated luminal specific p53 pathway genes (N = 24) was tested for association with survival post brain metastasis (SPBM) using survival analysis methods. High vs low expression of the luminal specific p53 pathway gene NEFM was significantly associated with worse SPBM (log-rank test; p-value = 0.014).

**Discussion**

This study suggests that NEFM may be used as a potential prognostic marker for breast cancer brain metastasis.

### P26 Magnetic Resonance Spectroscopy Studies in Eating Disorders: A Systematic Review

#### Megan A.M. Chiasson^1^, Rose M. Swansburg^2^, Frank P. MacMaster^2^, Kayla D.Stone^2^

##### ^1^Royal College of Surgeons in Ireland, Dublin Ireland, ^2^Departments of Psychiatry and Pediatrics, University of Calgary, Calgary Canada, Department of Medicine

###### **Correspondence:** Megan A.M. Chiasson

**Introduction**

Eating disorders affect approximately 4% of Canadians and are the deadliest of all psychiatric disorders. However, current treatment approaches are lacking in efficacy, as the neurobiological underpinnings of eating disorders are poorly understood. Magnetic resonance spectroscopy (MRS) involves a non-invasive evaluation of cerebral neurochemistry using a conventional MRI scanner. This technique measures the neurometabolite levels in the brain. Abnormal concentrations of these metabolites are found to cause issues with brain function, often giving rise to psychiatric symptoms. However, a clear neurochemical pattern in eating disorders has not yet emerged. Therefore, the purpose of this study was to complete a systematic review of neurochemical brain imaging research in eating disorder populations.

**Methods**

The study protocol was registered in the International Prospective Register of Systematic Reviews (PROSPERO) database. Studies comparing MRS outcomes between eating disorder participants and healthy controls were included. Six databases (Medline, EMBASE, PsycINFO, CINHAL, Scopus, and Web of Science) were searched using combinations of terms including “eating disorder” and “magnetic resonance spectroscopy”.Data extracted from each study included (1) MRS method, (2) neurometabolite concentrations, (3) brain region investigated, (4) diagnoses, (5) illness history, (6) medication use, and (7) psychological assessments. The Preferred Reporting Items for Systematic Reviews and Meta-Analyses (PRISMA) guidelines were followed.

**Results**

11 studies were included (all deemed low risk of bias), investigating a total of 184 eating disorder patients, 21 recovered patients, and 197 healthy controls. All studies included anorexia nervosa participants. Two studies also included bulimia nervosa participants. The results were heterogeneous, with various brain regions and neurometabolites measured.

**Conclusion**

Given the diverse nature of the results, this systematic review identifies the need for further studies to increase understanding of the neurochemical profile of people with eating disorders. This may improve early detection of the disorder and inform future treatment approaches.

### P27: Medium Term Follow-up after Receiving Autologous Conditioned Plasma (ACP) Injectionin Patients with Knee Osteoarthritis (OA); Retrospective Study

#### Mosleh Alalyani^1^, Breandán Long^2^

##### ^1^4th med student at RCSI, ^2^Orthopaedic surgeon at Aut Even Hospital, Kikenny & Bon Secours Hospital, Limerick

###### **Correspondence:** Mosleh Alalyani

**Background**

Current clinical literature includes many papers evaluating Autologous Conditioned Plasma (ACP) therapy and describes it as promising (Wilson J., 2015). However, the majority of those papers focus on the therapy's potential to treat tendinopathy. Therefore, we aimed to assess the clinical effectiveness of ACP injection in treating cartilage degeneration in some knee osteoarthritis (OA) patients in Kilkenny, Ireland.

**Methods**

We surveyed a group of patients who suffered knee cartilage lesions and received ACP injections during the last two years (2018-2020). A total of 28 patients out of 32 participated in this study by completing an online questionnaire. Only patients diagnosed with knee OA, who were treated with ACP injection using the Arthrex Double-syringe technique and performed by the same surgeon, were included. **Results:** Based on a comprehensive survey, we analysed the response data from 28 knee OA patients. 15 subjects reported knee pain reduction after ACP injection, whereas 13 did not have the desired therapeutic effect. Also, the duration and degree of therapeutic effect varied.

**Discussion**

Our findings correspond with previous studies confirming ACP injection is still a clinically viable treatment in inflammatory and degenerative diseases. Even though ACP therapy has produced significant outcomes in over 50% of our patients for over a year, some other patients found the therapy not effective. This research has provided more data on ACP from knee OA patients, which could be utilised to broaden our understanding of the efficacy of ACP injection.

**Keywords:** Knee pain, Knee osteoarthritis, Autologous Conditioned Plasma injection, Cartilage degeneration.

### P28: Meta-Analysis Of Dual Antiplatelet Therapy Of Clopidogrel Plus Aspirin Versus Aspirin Monotherapy In Patients With Coronary Artery Disease

#### Haidar Ali Hamzah, Putri Mahirah Afladhanti, Muhammad Despriansyah Romadhan

##### Universitas Sriwijaya, Indonesia

###### **Correspondence:** Haidar Ali Hamzah

Aspirin is the most commonly used for treatment in patients with coronary artery disease (CAD). However, there are many evidences from several trials suggested that dual antiplatelet therapy (DAPT) might exhibit a better outcome and more effective than aspirin monotherapy. Thus, we aimed to assess the effect of DAPT versus aspirin monotherapy in patients with CAD. Electronic databases were performed in PubMed, EMBASE, and Cochrane from January 2005 to March 2021. We searched for randomized control trials comparing DAPT versus aspirin monotherapy in patients with CAD. Pooled effects estimates were reported as an odds ratio (OR) with 95% confidence intervals (CI) and calculated using random-effects model. RevMan 5.4 software was used for data analysis. Five randomized control trials with a total of 8,203 participants met the inclusion criteria. DAPT was found to have an association in reducing the risk of major adverse cardiovascular events (OR 0.72; 95% CI 0.57-0.90; p = 0.003; I2 = 17%) and death events (OR 0.62; 95% CI 0.49-0.79; p < 0.0001; I2 = 0%) compared with aspirin monotherapy. Yet, there were no significant difference in myocardial infarction events (OR 0.78; 95% CI 0.57-1.07; p = 0.13; I2 = 28%) and bleeding events (OR 1.65; 95% CI 0.97-2.81; p = 0.06; I2 = 0%) between two groups. DAPT treatment has a significant effect in reducing the risk of major cardiovascular and death events without a significant effect in myocardial infarction and bleeding events compared with aspirin monotherapy.

### P29: Microstructural changes along the cingulum in young adolescents with psychotic experiences: an along-tract analysis

#### Areej Gazzaz^1^, Darren Roddy^2^, Elena Roman^1^, Anurag Nasa^2^, Vitallia Sooknarine^1^, Mary Cannon^1^

##### ^1^Royal College of Surgeons in Ireland, Ireland, ^2^Trinity College Dublin, Ireland

###### **Correspondence:** Areej Gazzaz

The cingulum is a discrete white matter tract beneath the cingulate cortex and it connects the overlying frontal, parietal, cingulate, and temporal regions with roles in memory, attention and emotional processing. Psychotic Experiences (PEs), such as hallucinations or delusions, have been shown to be particularly common among young people. Children or adolescents that are younger (9-12 years) have a higher prevalence of PEs compared to older (13-18 years) adolescents. Although, most young people who describe PEs in early life do not develop a later psychiatric illness, these individuals are at increased risk for a later diagnosis of a psychotic disorder such as schizophrenia. Further neurobiological research in these vulnerable young people may aid our understanding of how psychosis develops. We hypothesised that cingulum differences exist in young people with PEs not at the level of the four cingulum sections, but rather at the more granular voxel sized measurements. Whole brain High Angular Resolution Diffusion Imaging (HARDI) of 25 young people aged 11-13 years with PEs and 25 young people that were matched for age, sex, and handedness, without psychotic experiences were performed. No differences were found at the level of cingulum sections; however, region specific clusters of microstructural change were found in the left retrosplenial and right subgenual areas using along-tract analysis. This is the first study to use both section and along tract analysis in young adolescents with PEs, and the first study to show that along tract analysis may be useful in uncovering deeper, previously hidden, differences in the cingulum.

### P30: Multi-omic profiling of patient pancreatic cyst fluid for the identification of a novel biomarker panel of patient cancer risk

#### Laura E. Kane^1^, Gregory S. Mellotte^2^, Simone Marcone^3^, Paul F. Ridgway^2^, Kevin C. Conlon^3^, Barbara M. Ryan^2^, Stephen G. Maher^3^

##### ^1^Trinity College Dublin, Ireland, ^2^Department of Gastroenterology, Tallaght University Hospital, Dublin 24, Ireland, ^3^Department of Surgery, Trinity St. James’s Cancer Institute, St. James's Hospital, Dublin 8, Ireland

###### **Correspondence:** Laura E. Kane

**Introduction**

Pancreatic cancer was responsible for almost 500,000 deaths globally in 2020 according to GLOBOCAN 2020. Pancreatic cystic lesions (PCLs) are fluid-filled protrusions either on or inside the pancreas and can either be benign or pre-malignant. Current guidelines to stratify patients based on risk are imperfect. Multi-omic profiling of the fluid within pancreatic cysts could aid in the identification of a novel biomarker panel of patient cancer risk.

**Methods**

Patient pancreatic cyst fluid (PCF) was collected from 40 patients by EUS-FNA. Patients were stratified using the 2018 European evidence-based guidelines into low-risk (n=15), high risk (n=15) and no-risk or pseudocyst (n=10). PCF was briefly sonicated and subsequently processed using an SP3 paramagnetic bead protocol prior to LC-MS. MS-generated label-free quantification intensity data were analysed in Perseus (v1.6.13.0). HTG microRNA whole transcriptome sequencing was run on whole PCF. MiRNA sequencing data were analysed using HTG EdgeSeq Reveal (v3.1.0).

**Results**

MS-analysis of PCF samples revealed eight proteins to be significantly upregulated in high-risk PCF compared to low-risk (p<0.05, FDR=0.05, s0=0.1). Among the eight proteins identified, seven have been shown to be upregulated in pancreatic cancer cells. Whole transcriptome sequencing revealed forty-six miRNAs were significantly upregulated in high-risk PCF compared to low-risk (adj-p<0.05, FDR=0.05, s0=0.1). Five of the identified miRNAs are known to be upregulated in pancreatic ductal adenocarcinoma (PDAC) tissues and three have been shown to be upregulated in the circulation of PDAC patients. Differentially expressed proteins and miRNAs are currently being utilised to create an integrated, multi-omic predictive algorithm for low- and high-risk patients.

**Conclusion**

Multi-omic profiling of pancreatic cyst fluid provides an abundance of potential biomarkers that could be utilised independently or as part of a multi-marker panel for the stratification of patients into high-and low-risk groups for malignancy.

### P31: Nsaids And Covid-19: Do They Hit It Off?

#### Andra Diana Tanasa, Alice Colescu, Alexandra Condurat

##### UMF IASI Romania

###### **Correspondence:** Andra Diana Tanasa and Alice Colescu

**Background**

In the incipient phase of the pandemic, it was suggested by many healthcare officials that the use of NSAIDs (non-steroidal anti-inflammatory drugs) could affect both the risk of infection and the severity of COVID-19 and its related outcomes. This is due to the fact that NSAID treatment leads to upregulation of angiotensin-converting enzyme 2 (ACE2), the cell entry receptor for SARS-CoV-2, thus raising concerns that NSAIDs could increase susceptibility to infection. We analyze the data regarding the association between NSAIDs and COVID-19.

**Methods**

Studies focusing on the correlation between NSAIDs and COVID-19 were selected with the help of the medical search engine PubMed and included in a meta-analysis, with the help of formulas: hazard ratios, odds ratios, confidence intervals. These papers described the following: NSAID exposure and the risk of SARS-CoV-2 positivity, the risk of hospital admission in positive patients, exposure to ibuprofen and death, severe outcomes (ICU, ventilation or death).

**Results**

Studying and scanning the papers analyzing this issue, it was revealed that in SARS-COV-2-positive patients that were exposed to NSAIDs were not associated with higher risk of hospital admission ( OR 0.90, 95% CI 0.80–1.17 ), death (OR 0.88, 95% CI 0.80–0.98 ), severe outcomes (OR 1.14, 95% CI 0.90–1.44 ). There was no excess risk of SARS-CoV-2 positivity (OR 0.86, 95% CI 0.71–1.05 ). The acute use of ibuprofen was not associated with a greater risk of mortality -adjusted HR 0.632- (95% CI 0.073-5.441). The chronic use of NSAID use was also not associated with a greater risk of mortality -adjusted HR 0.492- (95% CI 0.178-1.362).

**Discussion**

The use of Ibuprofen and other NSAIDs in COVID-19 infected patients did not lead to worse COVID-19 outcomes. The use of NSAIDs did not increase the risk of SARS-COV-2 positivity.

### P32: Prevalence of myocarditis and cardiomyopathies in cardiology in-patient department

#### Denis Ruchkin, Anna Nartova, Anna Zaitseva, Yulia Lutokhina, Olga Blagova

##### First Moscow State Medical University, Russian Federation

###### **Correspondence:** Denis Ruchkin

**Introduction**

The prevalence of myocarditis and cardiomyopathies among cardiac patients is unknown. This study was aimed to determine it.

**Methods**

The study included 671 cardiology department patients. Case histories were randomly selected out of the total number of patients hospitalized between 08.07.2011 and 02.12.2019.

**Results**

Myocarditis was diagnosed in 28.9% (n=194) of the patients. Patients with myocarditis were significantly younger than others: 50.55±14.6 years vs 63.33±16.18 years, p < 0.001, 60.8% were male. Phenotypes of myocarditis included arrhythmic (47.4%, n=92), dilated cardiomyopathy (DCM) phenotype (41.2%, n=80), ischemic (8.3%, n=16), infarction-like (1.6%, n=3), latent (1%, n=2) and Loeffler endomyocarditis (0.5%, n=1). The diagnosis of cardiomyopathy was made in 76 patients: left ventricular noncompaction (LVNC) — 39.5% (n=30), DCM — 17.1%(n=13, in 8 primary genetically determined, in 5 patients — toxic etiology), hypertrophic cardiomyopathy (HCM) — 13.2%(n=10), arrhythmogenic right ventricular cardiomyopathy (ARVC) — 11.8%(n=9) and restrictive cardiomyopathy — 6.6%(n=5). The other cardiomyopathies were present in 11.8%(n = 9) of the cases: ischemic cardiomyopathy — 4%(n = 3), cardiomyopathy as a manifestation of storage diseases — 2.6%(n = 2), the combination of LVNC and ARVC — 2.6%(n = 2), the combination of LVNC and HCM — 1.3%(n = 1) and anthracycline cardiomyopathy — 1.3%(n=1). Superimposed myocarditis was observed in 34.2% (n=26) of patients with cardiomyopathies: with LVNC — 53.3%(n=16), with ARVC — 77.8%(n=7), with primary DCM — 12.5%(n=1) and 40%(n=2) of patients with DCM of a toxic etiology.

**Discussion**

The data suggests a high incidence of myocarditis among cardiology department patients. In practice myocarditis is often missed due to nonspecific manifestations. Patients with rhythm abnormalities and DCM syndrome should be screened for myocarditis. Cardiomyopathies are often combined with myocarditis, which should also be actively identified in these patients. A limitation of this study — the sampling error in a random selection of the medical cards.

### P33: Profiling the immune component of pancreatic cyst fluid to identify novel biomarkers of patient cancer risk

#### Laura E. Kane^1^, Gregory S. Mellotte^2^, Rebecca G. Lyons^1^, Eimear Mylod^1^, Simone Marcone^1^, Paul F. Ridgway^2^, Kevin C. Conlon^1^, Barbara M. Ryan^2^, Joanne Lysaght^1^, Stephen G. Maher^1^

##### ^1^Department of Surgery, Trinity St. James’s Cancer Institute, St. James's Hospital, Dublin 8, Ireland, ^2^Department of Gastroenterology, Tallaght University Hospital, Dublin 24, Ireland

###### **Correspondence:** Laura E. Kane

**Background**

Pancreatic cancer has a 5-year survival rate of less than 5%. Pancreatic cystic lesions are fluid filled sacs found within or on the surface of the pancreas, which can be either benign or pre-malignant. Current guidelines used to stratify patients into high- and low-risk of malignancy are imperfect, heavily debated. Pancreatic cyst fluid (PCF) has been shown to contain many inflammatory proteins, which have the potential to differentiate low- and high-risk cysts.

**Methods**

PCF was collected from 40 patients by EUS-FNA. Patients were stratified using the 2018 European evidence-based guidelines into low- and high-risk groups. PCF was sonicated and subsequently processed using SP3 paramagnetic beads prior to LC-MS. MS-generated data were analysed in Perseus (v1.6.13.0) and STRING (v11.5). Flow cytometry analysis was performed on the PCF cell pellet and analysed using FloJo (V10).

**Results**

Flow cytometry analysis revealed no lymphocyte population in the PCF. MS-analysis of PCF revealed a total of 465 proteins present in a minimum of 6 samples. STRING analysis revealed 55 and 32 proteins to be involved in the innate and adaptive immune responses, respectively. Significantly positive correlations were found between 10 proteins and patient risk classification (p<0.05). Differential expression analysis revealed 3 of these proteins to be significantly upregulated in high-risk PCF compared to low-risk (p<0.05, FDR=0.05, s0=0.1). Differentially expressed proteins are currently being technically validated by ELISA. They will then be examined in matched patient serum with the aim of generating a less invasive predictive biomarker panel for patient risk.

**Conclusions**

PCF contains an abundance of immune relevant proteins. Further interrogation is required to determine the origin of these proteins, as no lymphocyte population could be identified. Several of these secreted proteins are differentially expressed between low- and high-risk patients and have the potential to be utilised as novel biomarkers of patient risk.

### P34: Pulmonary embolism risk stratification in cardiovascular patients and its preventive measures

#### Hossam Bajbouj, Anna Tytova

##### Kharkiv National Medical University Ukraine

###### **Correspondence:** Hossam Bajbouj

**Introduction**

The prevalence of pulmonary embolism is estimated to be 60-70 per 100,000, Nevertheless, actual numbers are exponentially higher due to silent PE in 40%-50% of cases. The study focused on the association between cardiovascular disorders and PE.

**Methods**

The study population consisted of 58 patients with cardiovascular pathology (37 female and 21 male). To estimate the risk of pulmonary embolism in cardiovascular patients; PE clinical score was used: Geneva scale and Wells scale.

**Results**

According to the risk of developing PE, patients were divided into 3 groups. The 1st group –low risk– 12 patients (5 men and 7 women,56,8±3,4 years of age), among them 42% with atherosclerotic cardiosclerosis, 25% with stable angina and 33% with NQMI, the 2nd group -intermediate risk– 28 patients (10 men and 18 women, 62,5±4,6 yrs.): 53% had stable angina, 7% unstable angina, 10% QMI, 20% QMI and 10% atherosclerotic cardiosclerosis; 3rd group –high risk- 9 patients ( 4 men and 5 women, 64,6±6,7 yrs.) 14% stable angina, 14% unstable angina, 14% QMI and 58% atherosclerotic cardiosclerosis. It was found that complications such as atrial fibrillation, extrasystolic arrhythmia, COPD, chronic bronchitis, and autoimmune thyroiditis increase the risk of PE in patients with cardiovascular diseases. The high and intermediate-risk groups were mostly seen in patients with NQMI and QMI. However, the highest number of cases with intermediate-risk were among patients with stable angina and atherosclerotic cardiosclerosis; also, atherosclerotic cardiosclerosis patients showed a 58% of high risk of PE. This confirms propitious anticoagulant therapy in patients with unstable angina, NQMI, and QMI, but insufficient anticoagulation in patients with stable angina and atherosclerotic cardiosclerosis. **Discussion:** Based on the above-mentioned data, more intensive anticoagulant/antiplatelet therapy should be introduced into treatment protocols of patients with stable angina and atherosclerotic cardiosclerosis due to their higher risk of developing PE.

### P35: Quinolone Prescribing in Tallaght University Hospital: 2020 Point Prevalence Study

#### Asten Yeo^1^, Hannah McElvaney^1^, Tom Ragonetti^1^, Anna-Rose Prior^2,3^

##### ^1^School of Medicine, Trinity College Dublin, the University of Dublin, Ireland, ^2^Department of Clinical Microbiology, Tallaght University Hospital, Dublin, Ireland, ^3^Department of Clinical Microbiology, Trinity College Dublin, the University of Dublin, Ireland

###### **Correspondence:** Asten Yeo

**Introduction**

Quinolones are a class of broad-spectrum antibiotics that have been used to effectively treat a wide variety of infections. However, recent recommendations have restricted quinolone prescribing due to concerns of potentially severe side effects; current guidelines state they should only be prescribed in cases of significant penicillin allergy or as a first-line treatment in a few specific infections. The purpose of this study was to (1) assess the prevalence of quinolone prescribing in Tallaght University Hospital (TUH), (2) analyze the indications for their prescription and adherence to hospital guidelines, (3) identify patients with risk factors that increase their susceptibility to adverse effects, and (4) assess the proportion of patients suitable for an intravenous-to-oral switch.

**Methods**

A standardised survey form was used to collect data from patient drug charts, medical charts, and nursing notes over a 96-hour period between 05–08 October 2020. All adult inpatients with an active quinolone prescription were included, with the exception of patients in the emergency department. Data was evaluated using simple descriptive statistics.

**Results**

2.2% (9/404) of adult inpatients at TUH had an active prescription for fluoroquinolones, with 7/9 patients prescribed ciprofloxacin and 2/9 patients prescribed levofloxacin. 2/9 patients were prescribed fluoroquinolones incongruent with TUH guidelines. 8/9 patients had at least one identified risk factor that increased their susceptibility to fluoroquinolone side effects. 1/9 patients were suitable for an intravenous-oral switch.

**Discussion**

Special considerations should be taken when prescribing quinolones outside of first-line indications, consulting the TUH microbiology department should be considered in these instances. Thorough history taking and the accurate charting of the severity of penicillin allergies may reduce the inappropriate prescribing of quinolones.

### P36: REDD1 (Regulated in development and DNA damage 1) inhibition for safer glucocorticoid-based therapy of breast cancer

#### Evgeniya Lylova^1^, Darya Demina^2^, Ekaterina Lesovaya^1^

##### ^1^N.N. Blokhin National Medical Research Center of Oncology Russian Federation; ^2^MIREA — Russian Technological University Russian Federation

###### **Correspondence:** Evgeniya Lylova

**Introduction**

Glucocorticoids (GCs) are widely used as adjuvants in the therapy of breast cancer (BC). Unfortunately, chronic treatment with glucocorticoids commonly leads to serious adverse effects including skin and muscle atrophy and osteoporosis. We found recently that REDD1 (regulated in development and DNA damage 1) plays central role in the development of GC-induced side effects as well as with tumor malignization and progression in several cases. Here we tested whether REDD1 suppression makes glucocorticoid-based therapy of breast cancer safer. The aim of the present work is the screening of REDD1 inhibitors on BC cell lines of luminal and triple negative subtypes.

**Methods**

Putative REDD1 inhibitors were selected by bioinformatical screening. Triple negative (MDA-MB-231) and luminal (MCF7) BC cell lines were cultivated with putative REDD1 inhibitors Apigenin, Resveratrol, Curcumin, Emetin, CGP60474, Rapamycin, Wortmannin, LY294002 individually and in combination with glucocorticoid Dexamethasone. Expression of REDD1 and GC-regulated gene FKBP51 was evaluated by quantitative PCR, level of REDD1 protein and phosphorylated glucocorticoid receptor (GR) was evaluated by Western blotting.

**Results**

All compounds except Emetin inhibited basal and GC-induced REDD1 expression in both cell lines on mRNA level. REDD1 protein expression was suppressed by Resveratrol, CGP60474, Wortmannin, Rapamycin and Apigenin. The expression of FKBP51 was inhibited by all studied compounds.

**Discussion**

We demonstrated the inhibition of the basal and GC-induced REDD1 expression on mRNA and protein level by CGP60474, Rapamycin, Wortmannin, Resveratrol and Apigenin. Expression of FKBP51, marker of GR dimerization and GC resistance, also was suppressed, Therefore, the selected compounds could be used for the development of safer GC-based BC treatment protocols. Work was supported by Russian Science Foundation grant 17-75-20124

### P37: Resting Metabolic Rate (RMR) and Its Relation to Anthropometric Parameters and Glycemic Control Type 2 Diabetes Mellitus Patients - A Secondary Data Analysis

#### Geetah Rajandrin^1^, Jeevan K Shetty^2^, Prabhath M^3^, Arun G Maiya^3^, Shashikiran Umakanth^4^

##### ^1^Perdana University - Royal College of Surgeons in Ireland, Malaysia, ^2^Manipal University College Malaysia (MUCM), Melaka, Malaysia, ^3^Department of Physiotherapy, School of Allied Health Sciences, Manipal Academy of Higher Education, India, ^4^Dr TMA Pai Hospital (Udupi) and Melaka Manipal Medical College (Manipal Campus), Manipal Academy of Higher Education, India

###### **Correspondence:** Geetah Rajandrin

Type 2-diabetes Mellitus(T2DM) is an increasingly prevalent health condition worldwide. It is also associated with metabolic alterations due to insulin deficiency and resting metabolic rate (RMR) changes have been reported. Present secondary data analysis was designed to evaluate the pattern of RMR in T2DM male and female patients and to explore its relation to anthropometric parameters and HbA1c in coastal Karnataka, India. This is secondary data analysis from a cross-sectional study having 467 subjects with T2DM. The study recruited 157 T2DM patients taking oral medications, regular follow up and free of DM related complications. Details on patient's demographics (age & duration of disease), HbA1c, anthropometric data (weight, height, BMI, waist circumference) and body fat indices (RMR & visceral fat) were obtained. The RMR and anthropometric measurements of the male and female were compared with the reference values using a one-sample t-test. RMR was correlated with waist circumferences, visceral fat, BMI and HbA1c using Pearson correlation. The waist circumference and HbA1c were significantly higher compared to the recommended values in both males and female T2DM patients. The BMI was significantly higher in females compared to normal reference values. The RMR was significantly lower in both males and females compared to normal reference values. There was a significant positive correlation between RMR and waist circumference (p<0.001 & r=0.294), BMI (p<0.001 & r=0.440), and visceral fat (p<0.001, r= 0.534). There was a negative correlation observed between RMR and glycated Hb (p=0.269, r=-0.089), which was statistically not significant. We relate the lower RMR in T2DM patients in our study group to low visceral fat and long-term glycaemic control as reflected by their HbA1c levels. In addition to medications and diet, measuring RMR and anthropometric parameters can complement to achieve the goal of T2DM management.

### P38: Safety and Efficacy of Anti-TNFα Therapy in Inflammatory Bowel Disease Patients With NYHA III-IV Heart Failure: A Systematic Review and Meta-Analysis

#### Talal Almas^1^, Maryam Ehtesham^1^, Muhammad Ali Niaz^1^, Absam Akbar^2^, Omer Khan^1^, Gregory Cooper^3^, Emad Mansoor^3^

##### ^1^Department of Medicine, RCSI University of Medicine and Health Sciences, Dublin, Ireland, ^2^Aga Khan University, Karachi, Pakistan, ^3^Division of Gastroenterology and Liver Disease, University Hospitals Cleveland Medical Centre, Case Western Reserve University, Cleveland, OH, USA

###### **Correspondence:** Talal Almas

**Introduction**

Tumour necrosis factor (TNF)-alpha has been widely implicated in inflammatory bowel disease (IBD), with anti-TNF biologics constituting an integral component of IBD pharmacotherapy. Nevertheless, their uptake in patients with concomitant heart failure has been linked to worsening prognosis. Consequently, anti-TNF therapy remains contraindicated in IBD patients with class III-IV New York Heart Association (NYHA) heart failure.

**Methods**

In accordance with the PRISMA guidelines, we queried the Cochrane, PubMed, and Clinicaltrials.org databases from inception till 2020 using the medical subject headings (MeSH) “inflammatory bowel disease”, “anti-TNF”, “heart failure”, and “disease modifying anti-rheumatic drugs”. Studies that reported the adverse cardiovascular effects of anti-TNFα therapy in IBD patients compared to conventional anti-inflammatory therapy were included in the analysis.

**Results**

The search yielded a total 10 results, of which three randomised controlled trials, comprising 219 patients with stable class III-IV NYHA heart failure were identified. Anti-TNFα therapy in IBD patients was associated with increased odds of exacerbation and decompensation (Hazard ratio: 1.23, 95% confidence interval: 1.11-1.46; P < 0.05; I2 =23%). Importantly, no differences in left ventricular function were noted at the study end-point.

**Discussion**

In IBD patients with stable class III-IV NYHA heart failure, anti-TNFα therapy was associated with increased odds of decompensation and acute heart failure exacerbations. Anti-TNFα therapy within this patient cohort should therefore be employed with due caution. Further trials are needed to better elucidate this ostensible link.

### P39: Safety and Efficacy of Sodium Glucose Co-transport 2 Inhibitors in Heart Failure Patients Treated With Sacubitril-Valsartan: An Updated Meta-Analysis

#### Talal Almas^1^, Muhammad Ali Niaz^1^, Maryam Ehtesham^1^, Absam Akbar^2^, Omer Khan^1^ David Song Icahn^3^ Aamir Hameed^4^

##### ^1^Department of Medicine, RCSI University of Medicine and Health Sciences, Dublin, Ireland, ^2^Aga Khan University, Karachi, Pakistan, ^3^School of Medicine at Mount Sinai - Elmhurst Hospital Center, Elmhurst, NY, USA, ^4^Tissue Engineering Research Group (TERG), Department of Anatomy and Regenerative Medicine, RCSI University of Medicine and Health Sciences, Dublin

###### **Correspondence:** Talal Almas

**Introduction**

In recent times, there has been mounting fervour regarding the efficacy of sodium-glucose cotransport 2 inhibitors (SGLT2is) in patient with heart failure. Recent trials have divulged a reduction in overall all-cause and cardiovascular mortality within the treated cohort. Nevertheless, data evaluating the efficacy of SGLT2is in heart failure patients treated with a sacubitril-valsartan regimen remains sparse.

**Methods**

We perused the MEDLINE, Cochrane, SCOPUS, and EMBASE databases using the medical subject headings (MeSH) terms “sacubitril-valsartan”, “heart failure”, “sodium glucose co-transport 2 inhibitors” and “mortality” from inception to date. The primary outcomes were heart-failure hospitalisations and associated mortality. A Cox proportional hazard model was employed, and the heterogeneity within the sample sizes was ascertained using the Higgins I2 statistic (I2 > 50% was considered significant). These data were then pooled for the present meta-analysis.

**Results**

The initial literature search yielded 932 articles. Of these, 4 randomised controlled trials were identified, yielding a total study population comprising 1026 subjects. When pooled together, the analysis revealed an overall reduction in mortality in HF patients treated with a dual sacubitril-valsartan and SGLT2i regimen (Hazard ratio: 0.82, 95% CI: 0.74-0.89, P<0.05).

**Discussion**

While the data pertaining to the uptake of a dual regimen involving sacubitril-valsartan and SGLT2i remains sparse, this meta-analysis should form the basis of further clinical trials within the area and could portend favourable outcomes in HF patients.

### P40: Septicaemia due to Elizabethkingia meningoseptica: An emerging and rare pathogen.

#### Dhruv Qureshi^1^, Shahzad Mirza^1^, Nikunja Das^1^, Sahjid Mukhida^1^, Sriram Kannuri^1^

##### ^1^Dr. D.Y. Patil Medical College, Hospital and Research Centre, Dr. D. Y. Patil Vidyapeeth, India

###### **Correspondence:** Dhruv Qureshi

**Introduction**

Elizabethkingia meningoseptica is a non-fermenting Gram-negative bacillus ubiquitously found in hospital environments, associated with Hospital-Acquired Infections. Immunocompromised individuals are particularly at increased risk for developing severe infections due to E. Meningoseptica, which is resistant to multiple classes of antibiotics. We report a series of septicaemia cases due to E. meningoseptica in patients admitted in Intensive Care Units (ICUs) who had bloodstream infections, and were onerous in treating them and required unconventional drugs for positive outcome.

**Methods**

Around 8-10 ml blood was aseptically collected in BacT/ALERT bottles from inpatients in Intensive Care Units and were incubated until a positive signal. Identification and antibiotic susceptibility testing was performed using VITEK 2C as per Clinical Laboratory Standards Institute Guidelines.

**Results**

We reported 3 separate cases of central line-associated blood stream infection in patients admitted in ICUs. All three blood cultures grew E. meningoseptica which was resistant to majority of classes of antibiotics. All three patients were clinically symptomatic even after treatment with higher classes of antibiotics like carbapenems. As per culture sensitivity reports and consultation with clinical microbiologists, the patients were started on Vancomycin and Teicoplanin. Two patients recovered well after timely receiving treatment while one patient didn’t show a favourable outcome.

**Discussion**

Studies and some literature reveal that E. meningoseptica is rare emerging pathogen resistant to most antibiotics used in treating gram-negative organisms, because it produces class-A extended spectrum β-lactamases and carbapenemases intrinsically, but responds well to fluoroquinolones, macrolides and cell wall-inhibiting drugs like vancomycin and teicoplanin, though the mechanism of action is not yet clearly understood. Aseptic precautions and hand hygiene play a major role in reducing the incidence of infection with this organism. Automated systems can play a major role in identification of such rare organisms, which play a key role in management of these cases.

### P41: Shielding advice and its association with mortality in the COVID-19 pandemic

#### Azmaeen Zarif^1^, Mark Joy^1^, Julian Sherlock^1^, Dylan McGagh^1^, Simon de Lusignan^1,2^

##### ^1^Nuffield Department of Primary Care Health Sciences, University of Oxford, United Kingdom, ^2^Royal College of General Practitioners United Kingdom.

###### **Correspondence:** Azmaeen Zarif

**Introduction**

In England, individuals deemed extremely likely to be vulnerable to COVID-19 infection were advised to self-isolate, termed ‘shielding’, for 12 weeks to prevent infection. We studied whether the people who received shielding advice reinforced by their GPs had a reduced mortality risk compared to a matched cohort not advised to shield.

**Methods**

We conducted a retrospective cohort study using a nationally representative sample comparing people aged >=40 years who were recorded as being advised to shield using a fixed ratio of 1:1, matching to people with the same diagnoses not advised to shield (n=77,360 per group). Time-to-death analysis was undertaken using Cox regression. A sensitivity analysis compared exact matched cohorts.

**Results**

We report the time-varying hazard ratio (HR) of mortality between groups. Mortality risk in people shielding was half compared to those not shielding (HR=0.50, 95%CI:0.41-0.59. p<0.0001) in the first three weeks. Over the remaining nine weeks, mortality risk was 54% higher in the shielded group (HR=1.54, 95%CI:1.41-1.70, p<0.0001). After cessation of the twelve-week shielding period, mortality risk was over two-and-a-half times greater in the shielded group (HR=2.61, 95%CI:2.38-2.87, p<0.0001).

**Discussion**

The recommendation to shield was associated with a short-term reduction in the risk of mortality in the shielded population, compared with matched controls. However, the protective effect lasted less than twelve weeks. The intervention as delivered across the first wave of the pandemic may have been insufficient. As shielding was not repeated in the subsequent waves of COVID-19 infection in England after the first lockdown, we are left with uncertainty about the role of shielding in future pandemics. More targeted shielding strategies, informed by additional holistic economic and psychological analyses regarding effective implementation to ensure maximal behavioural compliance may significantly reduce mortality risk as part of public health plans for future pandemics.

### P42: Social Determinants of Health in Trauma

#### Mrs Olivia Jagiella-Lodise^1^, Ashley Binder^2^, Randi Smith^3^

##### ^1^Royal College of Surgeons in Ireland, Ireland, ^2^University of Illinois, United States, Emory University School of Medicine, United States

###### **Correspondence:** Olivia Jagiella-Lodise

**Background**

The Adverse Childhood Experiences score (ACEs) is a tally of physical, emotional or sexual abuse that one may experience during childhood. Several chronic health conditions have been associated with high ACEs, however, the link between ACEs and traumatic injury in adulthood is not fully understood. Hence, the objective of this study was to explore the impact of ACEs and other contextual factors on outcomes after trauma.

**Methods**

We performed a retrospective chart review for a convenience sample of adult trauma patients at an urban Level 1 trauma center between September 2019 and March 2020. Demographics, injury characteristics, clinical outcomes and ACE scores were obtained and analyzed. ACEs were determined through self-report. High ACE scores were considered >= 4, consistent with current literature. Statistical significance was considered P < 0.5.

**Results**

108 subjects were included. The median age was 61 years, median ISS = 16 and the majority were male (n=76, 71%) and Black (n=75, 69%). Mean ACEs for the cohort = 3.5. ACEs did not correlate with injury severity (p=0.465) or ICU length of stay (LOS) (P=0.13, P=0.309). A high ACE score did correlate to a hospital LOS<5 days (P=0.03). An ACE score >=4 was not associated with a specific mechanism of injury (P=0.18) or substance abuse (P=0.32), but did correlate with psychiatric illness (P<0.01). Patients who had experienced violent ACEs specifically, were more likely to experience blunt force trauma (P=0.017). White race was associated with violent abuse (P=0.038) and neglect (P=0.029); while Black race was associated with witnessing someone being stabbed (P<0.01).

**Discussion**

Trauma patients have high ACEs. There were racial differences associated with ACEs, however, high ACEs did not correlate to worse outcomes. Perhaps, the similar outcomes amongst patients with low and high ACEs attest to equitable trauma care delivery at this single institution.

### P43: Testing the Efficacy of Targeted Small-Molecule Inhibitors in the Treatment of High Risk Multiple Myeloma

#### Katia Yazji^1^, Harmony Black^2^, Siobhan Glavey^1^

##### ^1^Royal College of Surgeons in Ireland, Ireland, ^2^National Health Service UK, United Kingdom

###### **Correspondence:** Katia Yazji

Multiple Myeloma (MM) is an aggressive plasma cell malignancy in which all patients eventually become resistant to treatment. Bortezomib, the standard of care for myeloma patients combined with Olaparib, a drug used in the treatment of breast/ovarian cancers, may prove to be a novel therapeutic regimen in the treatment of SKY92 high risk (HR) MM. We analyzed microarray data from MM samples in an elderly patient population using the MM Profiler™. The normalized cell line gene expression data was inputted into the SKY92 algorithm which yielded a risk-score for the sample, either HR or standard risk (SR). We selected 2 cell lines and performed in vitro dose response assays to explore the potential for therapeutic targeting of SKY92 genes. Cells were treated for 72hrs with varying concentrations of single agent Bortezomib and Olaparib. Cell viability was assessed at 24hrs, 48hrs and 72hrs using the CellTiter-Glo® assay. Dose response curves were plotted with GraphPad Prism. Combination drug treatments were performed in the same manner. Synergism between the two agents was calculated using the COMPUSYN software. SKY92 risk scores were calculated for all available MM cell lines using normalized cell line gene expression data. Two cell lines, JJN3, the 2nd highest risk line, and KMS18, the 22nd highest risk line, were chosen for dose response assays. Results showed that JJN3 cells were significantly less sensitive to Bortezomib than KMS18 cells at all single agent Bortezomib doses tested. The results of the viability assays for the combination treatment demonstrated that there was statistically significant JJN3 cell death at 48hrs (P=0.012) and 72hr (P=0.017) timepoints. Our findings show that SKY92 risk status is predictive of Bortezomib response in vitro. In addition, a combination treatment of Bortezomib and Olaparib may be a novel therapeutic regimen effective in enhancing cell death in HR MM.

### P44: The Clinical Value of Computed Tomography Coronary Angiography in Predicting Adverse Cardiac Events in Asymptomatic Patients With Established Coronary Artery Disease: A Systematic Review and Meta-analysis

#### Maryam Ehtesham^1^, Talal Almas^1^, Uzair Malik^1^, Omer Khan^1^, Kishor Vora^2^

##### ^1^Department of Medicine, RCSI University of Medicine and Health Sciences, Dublin, Ireland, ^2^Department of Cardiology, Owensboro Medical Practice, Owensboro, Kentucky, USA

###### **Correspondence:** Maryam Ehtesham and Talal Almas

**Background**

Coronary artery disease continues to be a leading cause of cardiovascular mortality globally. In asymptomatic patients with established coronary disease, a myriad of diagnostic modalities is routinely employed to ascertain the likelihood of major adverse cardiac events. Of these modalities, computed tomography angiography can be effectively utilised to predict the odds of future adverse cardiac events in the aforesaid cohort.

**Methods**

We searched the MEDLINE, PubMed, Embase, and Cochrane databases using a varied combination of medical subject headings that included “coronary artery disease”, “computed tomography”, and “ major adverse events”. The Preferred Reporting Items for Systematic Reviews and Meta-Analyses (PRISMA) was used to identify and shortlist the studies. Major adverse cardiac events such as myocardial infarction, arrhythmias, heart failure, and mechanical complications were studied across the studies. A Cox proportional hazard model was used. Furthermore, the heterogeneity in the sample included was determined using the Higgins I2 statistic (I2 > 50% was considered significant).

**Results**

Our comprehensive literature search yielded 12 articles. Articles in languages other than English were excluded, resulting in 10 articles overall. The overall sample size across the studies was 4912 patients, with their mean age hovering at 58.4 +/- 6.8 years (CI: 55.2- 68.6, P<0.05). Of the 3124 asymptomatic patients who underwent CT coronary angiography, 93.3%, amounting to 2915 patients, with remarkable CT angiography findings experienced an adverse major cardiac event at the 30-day period.

**Discussion**

The present meta-analysis revealed that in asymptomatic patients with established coronary artery disease, CT coronary angiography can be efficaciously employed to reliably predict the likelihood of major adverse cardiac events. These findings can have numerous clinical implications.

### P45: The Effect Of Body Composition On Renal Hemodynamic Parameters In Potential Male Kidney Donors

#### Milan Rodic, Teodora Novaković, Branislava Ilinčić

##### Medical Faculty, University of Novi Sad Serbia

###### **Correspondence:** Milan Rodic

**Introduction**

It is estimated that overweight people are up to three times more likely to suffer from end-stage kidney disease. This is mostly attributed to the most common comorbidities in this population such as hypertension and diabetes. However, some researchers suggest that obesity is a direct risk factor for the changes in kidney function. Moreover, effective renal plasma flow (ERPF) is commonly indexed to Body surface area (BSA), which can be misleading in people with abnormal body composition. Therefore the aim of the present study was to analyse effective renal plasma flow rate in healthy male potential kidney donors and determine potential correlation between effective renal plasma flow and body composition parameters such as body weight, fat mass, fat-free mass, body mass index, BSA and height.

**Methods**

The study was a retrospective analysis of data obtained from medical documents. The study group consisted of 39 healthy male potential kidney donors of average age 52.2±12.6 years. The analysis of laboratory blood sample results, radionuclid clearence of hippuran and anthropometric measurements has been performed.

**Results**

Median of Body mass index (BMI) was 27,5 kg/m2, which means that the majority of our examinees was overweight, with mean fat mass of 20,4% in total body weight. Mean rate of ERPF was 489,18 ml/min/1.73m2 which is on the average 13,6% less than expected for their age. In our study we established negative correlation between ERPF and both fat (r=-0,29, p=0,03) and fat-free mass (r =-0.39, p=0.02).

**Discussion**

Examined hemodynamic parameter ERPF was within expected values considering the age of men who were potential kidney donors. Both fat mass and fat-free mass correlated negatively with ERPF which goes in favor of thesis of renal hemodynamics change in obesity.

### P46: The Relationship Between Mean Corpuscular Volume and Vitamin B12 Level in a Canadian Population

#### Gerges Abdelsayed^1^, Joseph Girgis^1^, Nivin Azer^2^

##### ^1^Royal College of Surgeons in Ireland, Dublin, Ireland, ^2^Regent Primacy Medical Center, Canada

###### **Correspondence:** Gerges Abdelsayed

Introduction

Vitamin B12 (B12) is required by the body to synthesize red blood cells (RBC). B12 deficiency may lead to the development of megaloblastic anaemia, characterized by a decreased haemoglobin and increased mean corpuscular volume (MCV). This correlation is heavily emphasized in medical school curricula. However, reduced B12 level is not always correlated with increased MCV and vice versa. This may introduce issues when MCV is used as a diagnostic tool for B12 deficiency. This study aims to investigate the relationship between B12 deficiency and MCV in a Canadian cohort with asymptomatic B12 deficiency.

**Methods**

Using electronic medical records patients with B12 deficiency (n=83) were selected, along with controls (n=53). B12 levels of these patients were compared to their MCV levels at the time of diagnosis. Ethical approval was obtained. The data was analysed in STATA using logistic regression.

**Results**

The logistic regression model showed no significant association between MCV and B12 levels (OR:1.04, CI 0.98-1.12, p=0.175).

**Discussion**

These results show that MCV levels are not a reliable indicator of B12 deficiency. The heavy emphasis on the relationship between B12 levels and MCV in medical school curriculum may lead to confusion among medical students. Some primary care facilities still use MCV to screen for B12 deficiency. This method is inaccurate and may delay diagnosis’s, worsening patient outcomes. The relationship between MCV and B12 levels should continue to be a part of medical school curriculum, but students should be taught that elevated MCV is not a sensitive predictor of B12 deficiency.

### P47: The Way Bioinformatic Analysis Would Change The Diagnosis Of Maroteaux–Lamy Syndrome

#### Attila Tamás György, Zsolt Kovács

##### University of Medicine, Pharmacy, Science, and Technology of Târgu Mureș Romania

###### **Correspondence:** Attila Tamás György

**Background**

The diagnosis of lysosomal storage disorders is primarily based on enzyme assay and hence it could be challenging in most of the cases. This problem is well-illustrated by Mucopolysaccharidosis Type VI also known as Maroteaux–Lamy syndrome, in which the breakdown of glycosaminoglycans is damaged due to the deficiency in the enzyme arylsulfatase B (ARSB) caused by homozygous or compound heterozygous mutation in the ARSB gene (5q14). Particularly, due to ARSB disfunction dermatan sulfate and chondroitin sulfate molecules easily build up in cellular lysosomes, resulting in multi-organ affection.

**Objective**

The aim of our research is to ease the diagnosis of this rare autosomal recessive lysosomal storage disorder with the help of bioinformatic analysis. For this purpose, we are using a self-made program.

**Methods**

Using the results of a targeted DNA sequencing, a specific analysis of the ARSB gene can be performed with the help of the application we designed.

**Results**

The program written in C# is capable of recognizing the ARSB gene introduced in FASTA format and comparing it with the normal ARSB gene sequence, previously introduced. It can also determinate the exact positions and the type of nucleotides in which the introduced sequence differs from the normal one. In a further step the algorithm can transcribe the sequence of the introduced gene into mRNA, and even generate the structure of the protein encoded by this.

**Conclusion**

Bioinformatic processing provides a reliable and fast solution for early screening of Maroteaux-Lamy syndrome, because it recognizes any gene mutation and the necessary genetic sampling can be done within the first week after birth. In conclusion, this modern and cheaper procedure may be used in the future widely for the screening of Mucopolysaccharidosis Type VI, making it a well-appliable method for the future of precision medicine.

### P48: Travelling With Trauma: A Pilot Study Exploring How Distance Affects Patient Attendance at The Emergency Department in Rural Communities Surrounding CGMH

#### Gina Rizq^1^, Michael Lisi^2^

##### ^1^Royal College of Surgeons in Ireland, Dublin, Ireland, ^2^Collingwood General and Marine Hospital, Ontario, Canada

###### **Correspondence:** Gina Rizq

**Background**

Distance is a unique challenge that rural communities face when accessing emergency care. The rural lifestyle is not only known to have a high rate of accidental injuries but, is also coupled with an increased risk of death that is correlated with living further from a hospital.

**Objective**

This is a pilot study being conducted at Collingwood General and Marine Hospital (CGMH) to determine how distance affects patient attendance at the emergency department (ED). The aim is to determine if patients who live further from CGMH can prioritize their health as readily as people who live closer.

**Methods**

A literature search was conducted using databases such as Pubmed, the Cochraine library, and google scholar, compiling a total of (23) articles. The primary outcome will look at determining how many patients who visited the emergency department at CGMH came from distances of less than 10km, from 10-20 km and from greater than 20km. The secondary outcome will focus how many patients were admitted from each distance category. The results will be statistically analyzed using Stata or SPSS.

**Results**

It is anticipated that there will be higher percent of patients who are admitted to the emergency department who live father away in comparison to those of close proximity to CGMH.

**Discussion/Conclusion** The results could potentially push public health funding towards increasing access to care in rural communities and encourage the population to more readily seek access to health services when needed. Future research can further explore how patients decide when versus when not to come in the ER.

### P49: Traumatic Abdominal Wall Hernias As Indicators of Injury Severity and Need for Laparotomy

#### Mrs Olivia Jagiella-Lodise^1^, Jason Sciarretta^2^, Deepika Koganti^2^, Krystal Archer-Arroyo^2^, Elizabeth Benjamin^2^, S. Rob Todd^2^

##### ^1^Royal College of Surgeons in Ireland, Ireland, ^2^Emory University School of Medicine, United States

###### **Correspondence:** Olivia Jagiella-Lodise

**Introduction**

Traumatic abdominal wall hernias (TAWH) are uncommon, prompting their lack of management guidelines. Their prevalence is increasing due to improved access and quality of imaging studies. This study aimed to evaluate whether the presence of a TAWH was associated with intra-abdominal injury requiring emergent laparotomy in blunt force trauma patients.

**Methods**

The trauma registry of an urban level I trauma center was reviewed (1/2012 - 12/2020) for adult patients with blunt trauma diagnosed with a TAWH. Demographics, mechanism of injury, Injury Severity Score (ISS), BMI, hospital length of stay, TAWH size, type of repair, and outcomes were analyzed. Chi-Square, ANOVA single-factor, and two-tailed T-tests with descriptive statistics were performed. P< 0.05 was considered significant.

**Results**

38,749 trauma patients were admitted over the study period, with only 62 (0.16%) diagnosed with a TAWH. The most common TAWH was lumbar (82%). The majority of patients were male (60%); the median age was 35 years and an ISS of 20. The most common mechanism of injury was motor vehicle collision (58%). Seat location at the time of impact did not determine the hernia laterality (P=0.109). Increase BMI was associated with pelvic fractures (P=0.0241) and larger defect size (P=0.0314). Seatbelt sign (p=0.0455), defect size (p=0.0340), and ISS (p=0.000784) indicated a need for emergent laparotomy (51%). A minority of TAWHs (16%) were repaired at index operation. Failed expectant management was uncommon (10%). Overall mortality was 5%, with no deaths related to the hernia whether operative or nonoperative.

**Discussion**

TAWH were associated with other severe intra-abdominal injuries requiring emergent laparotomy. The results aligned with the notion that nonoperative repair of TAWH at index operation for other life-threatening injuries is safe. The outcomes did not differ between operative and nonoperative management; further investigation is needed to determine which patients benefit from TAWH repair at index operation.

### P50: Who Accesses Surgery in Sub-Saharan Africa? Evidence from Tanzania

#### Gerges Abdelsayed, Jakub Gajewski

##### Royal College of Surgeons in Ireland, Ireland

###### **Correspondence:** Gerges Abdelsayed

**Introduction**

Only 3.5% of the 234.2 million surgeries conducted each year are performed in the poorest third of the world, with Africa having the highest ratio of surgical disability-adjusted life-years per 1000 people. This research aims to analyze the types of procedures conducted in 3 hospitals in Tanzania, patients' age distributions, and the qualifications of the healthcare workers performing these surgeries.

**Methods**

Surgical data from January 2019 – August 2020 for three district hospitals was collected and analyzed using analysis of variance (ANOVA) tests to determine the differences in means.

**Results**

Of the total 2535 procedures performed, 1553 (61.3%) were obstetrics procedures, 537 (21.2%) were general surgical procedures, and the remaining 445 (17.5%) consisted of GI, male genitourinary, eye, ENT, and orthopedic procedures. All outcomes were comparable since all patients survived. Using ANOVA, the differences in means between age and procedure type were found to be statistically significant (p < 0.001). 51.7% of the procedures were emergencies, and 48.3% were elective. The 20–24-year-old age group had the most procedures performed (455). 1842 (77.6%) were performed by medical doctors (MD) and specialists, 531 (22.4%) were performed by assistant medical officers (AMO). General surgical procedures included herniorrhaphies (48%), laparotomies (22.7%), and others. 369 (68.7%) procedures were performed on men, and 168 (31.3%) on women, mostly in the 30–34-year-old group. 432 (80.4%) of these were performed by MDs and specialists, while 105 (19.6%) were by AMO's. 121 (22.5%) were emergencies and 408 (76%) were elective. This data may be used as a metric of healthcare capacity. For example, the ratio of emergent to total surgeries may be used as a measure of surgical capacity.

**Discussion**

In conclusion, analysis of hospitals in Tanzania has shown that most procedures performed are obstetrics related, mainly in the 20-24-year-old age group, and mainly performed by MDs.

### P51: Who Best Predicts Future Health Service Use? A mixed-methods prospective study in Primary Care

#### Mayura Loganathan^1^, Tharmegan Tharmaratnam^2^, Jason Nie^3^, Behnam Hashemi^4^, Shawn Tracy^4^, Ross Upshur^5^

##### ^1^Department of Family and Community Medicine, Temerty Faculty of Medicine, University of Toronto, Canada, ^2^Royal College of Surgeons of Ireland, Dublin, Ireland, ^3^Trillium Health Canada, ^4^Bridgepoint Collaboratory for Research and Innovation, Lunenfeld-Tanenbaum Research Institute, Sinai Health System, Toronto, ON, Canada, ^5^Dalla Lana School of Public Health, Lunenfeld-Tanenbaum Research Institute, Sinai Health System, University of Toronto Canada

###### **Correspondence:** Tharmegan Tharmaratnam

**Introduction**

Population aging is leading to an increasing incidence of frailty and associated negative health outcomes. The Frailty and Vulnerability Evaluation (FAVE) tool is a self-administered tool designed to capture medical, functional, and important social risks. We aimed to assess correlations between a patient’s complexity score (sum of the number of chronic conditions and medications), their own and their family physician (FP) ratings of current, future frailty, and vulnerability with future healthcare utilization.

**Methods**

Using a prospective, mixed-methods design, we surveyed 274 patients aged 65+ across 10 primary care practices in urban, suburban, and rural Ontario. Chart data was abstracted 1-year after FAVE administration to assess healthcare utilization, including FP and specialist visits (SP), labs, imaging, ER visits, and hospitalizations. In addition, chronic conditions and medications data were retrieved. FP judgements regarding their patients’ future frailty and vulnerability were obtained through physician surveys, with a chart audit at 1-year follow up, using composite outcome incidence (COI) (i.e., if patients experienced ER visit, hospitalization, and/or death). The primary outcome was the correlation of a patient’s complexity score (CS), their own and FP’s clinical judgments of frailty and future vulnerability, with 1-year healthcare utilization (COI).

**Results**

Preliminary data of 201/274 patients [mean age, 80.12 ± 6.67 years; 41% male; 59% female] demonstrated an average CS of 16.54. Physicians [MOU1] (n=178) who rated patient’s future functional risk as high, had the greatest COI (62.3%), while patients who rated their future health as poor demonstrated the greatest COI (57.5%). Additionally, increased clinical frailty was associated with a higher COI (57.1%).

**Discussion**

Our data demonstrates that patient and physician rating of clinical frailty and future health service utilization do not display significant incongruency. This may suggest patient and physician ratings along with clinical tools may be useful predictors of clinical frailty and overall healthcare utilization.

